# Unraveling the Molecular Mechanism of *S*-Nitrosation Mediated by *N*-Acetylmicroperoxidase-11

**DOI:** 10.1021/acs.inorgchem.3c00180

**Published:** 2023-03-30

**Authors:** Maria Oszajca, Angelika Jodłowska, Dorota Rutkowska-Zbik, Konrad Kieca, Grażyna Stochel

**Affiliations:** †, Faculty of Chemistry, Jagiellonian University, 30-387 Krakow, Poland; ‡ Polish Academy of Sciences, Jerzy Haber Institute of Catalysis and Surface Chemistry, 30-239 Krakow, Poland; § Doctoral School of Exact and Natural Sciences, Jagiellonian University, 30-348 Krakow, Poland

## Abstract

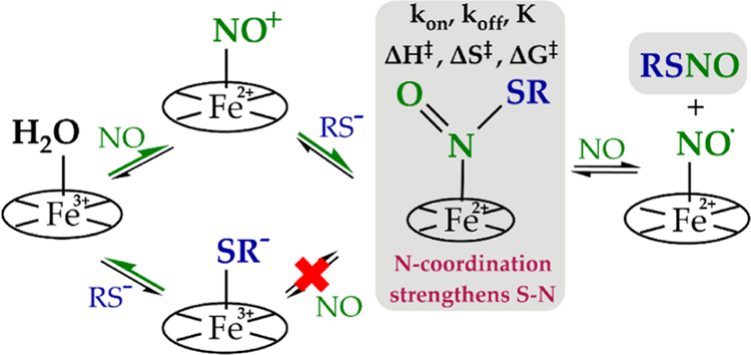

Conversion of NO to stable *S*-nitrosothiols is perceived as a biologically important strategy of NO storage and a signal transduction mechanism. Transition-metal ions and metalloproteins are competent electron acceptors that may promote the formation of *S*-nitrosothiols from NO. We selected *N*-acetylmicroperoxidase (AcMP-11), a model of protein heme centers, to study NO incorporation to three biologically relevant thiols (glutathione, cysteine, and *N*-acetylcysteine). The efficient formation of *S*-nitrosothiols under anaerobic conditions was confirmed with spectrofluorimetric and electrochemical assays. AcMP-11-assisted incorporation of NO to thiols occurs via an intermediate characterized as an *N*-coordinated *S*-nitrosothiol, (AcMP-11)Fe^2+^(N(O)SR), which is efficiently converted to (AcMP-11)Fe^2+^(NO) in the presence of NO excess. Two possible mechanisms of *S*-nitrosothiol formation at the heme-iron were considered: a nucleophilic attack on (AcMP-11)Fe^2+^(NO^+^) by a thiolate and a reaction of (AcMP-11)Fe^3+^(RS) with NO. Kinetic studies, performed under anaerobic conditions, revealed that the reversible formation of (AcMP-11)Fe^2+^(N(O)SR) occurs in a reaction of RS^–^ with (AcMP-11)Fe^2+^(NO^+^) and excluded the second mechanism, indicating that the formation of (AcMP-11)Fe^3+^(RS) is a dead-end equilibrium. Theoretical calculations revealed that *N*-coordination of RSNO to iron, forming (AcMP-11)Fe^2+^(N(O)SR), shortens the S–N bond and increases the complex stability compared to *S*-coordination. Our work unravels the molecular mechanism of heme-iron-assisted interconversion of NO and low-molecular-weight thiols to *S*-nitrosothiols and recognizes the reversible NO binding in the form of a heme-Fe^2+^(N(O)SR) motif as an important biological strategy of NO storage.

## Abstract 2

*N*-Acetylmicroperoxidase-11-mediated generation of *S*-nitrosothiols from NO and biologically relevant thiols (glutathione, cysteine, and *N*-acetylcysteine) was confirmed. Kinetic studies revealed that NO incorporation to thiols occurs in a reaction of (AcMP-11)Fe^2+^(NO^+^) with thiolate, whereas a direct reaction of (AcMP-11)Fe^3+^(RS) with NO does not yield the *S*-nitrosothiol. In the reaction pathway, an *N*-coordinated *S*-nitrosothiol, (AcMP-11)Fe^2+^(N(O)SR), was identified as a transient intermediate, which converts to (AcMP-11)Fe^2+^(NO) in the NO excess presence with the release of free *S*-nitrosothiol.

## Introduction

There are several mechanisms by which nitric oxide affects the biology of living cells. One of the best characterized is the binding of NO to iron heme, a protein cofactor, which results in the activation of soluble guanylyl cyclase (sGC)^1^ or inhibition of cytochrome *c* oxidase (CcO), a crucial enzyme for mitochondrial respiration.^2^ However, equally important are the *S*-nitrosation processes. So far, a large group of proteins that undergo *S*-nitrosation has been discovered, and the essence of this post-translational modification begins to be considered equally important to phosphorylation. *S*-Nitrosation is believed to regulate protein activity and function, thus playing an important role in cell function.^3,4^ It influences the functioning of receptors, transcription and translation factors, and calcium channels, among others. Furthermore, it plays an important role in redox signaling but is also attributed a protective role against oxidative stress. Low-molecular-weight *S*-nitrosothiols (GSNO and CysNO) and *S*-nitrosoproteins are reservoirs of NO, but they can also transfer NO^+^ and NO^–^. Despite the diverse role of *S*-nitrosothiols (RSNO) in biological systems, no coherent and efficient mechanism of their formation at the molecular level has been described in cells, although several reaction pathways leading to *S*-nitrosation have been proposed so far.^5^ Doctorovich et al.^6^ showed that, at anoxic conditions, NO and thiols react directly and in a double-step reaction form HNO and RSNO.^7^ While the formation of HNO has been confirmed, the quantity of RSNO generation has not been investigated by the authors. In the presence of dioxygen, NO oxidation to N_2_O_3_ is thought to be involved in the formation of *S*-nitrosothiols.^8^ Another proposed mechanism involves the oxidation of thiols by nitrogen dioxide (NO_2_) to thiyl radicals, which react with NO to give RSNO.^9^ However, the biological relevance of the pathways requiring the formation of N_2_O_3_ has been questioned due to the unfavorable kinetics under physiological conditions, and thus, it was suggested that they may be limited to a small subset of cellular compartments.^5,10^ It has been also shown that, under aerobic conditions at submicromolar NO concentrations, *S*-nitrosothiols are formed in a direct reaction of NO and thiols. Under such conditions O_2_ has been proposed to be an electron acceptor from a radical intermediate GSNOH^•^.^11^ Other mechanisms of physiological RSNO formation involving the participation of metal ions, especially bound in metalloproteins, and iron nitrosyl complexes have been highlighted.^12−14^ Metal ions (M^n+^: Cu^2+^, Fe^3+^) can act as electron acceptors either from a transient thionitroxyl radical (RSNOH^•^) oxidizing it to RSNO or from NO generating NO^+^, followed by a reaction with a thiol.^5^ Ferric-heme centers perceived as suitable electron acceptors that may satisfy the redox requirements necessary for the *S*-nitrosothiol (RSNO) synthesis are an area of intense research.^10,15−18^ Numerous proteins conduct auto-*S*-nitrosation catalyzed by their metal center, e.g., hemoglobin, cytoglobin, and neuroglobin.^19−21^ The mechanism proposed for both the auto-*S*-nitrosation of hemoglobin and the formation of *S*-nitrosoglutathione (GSNO) in the reactions of NO-mediated by myoglobin or ceruloplasmin involves the general reaction given in eq 1. The formation of a metal-nitrosyl intermediate with considerable M^(*n*-1)^–NO^+^ character is a prerequisite^18,22^
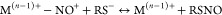
1

On the other hand, it was found that cytochrome *c* can promote the *S*-nitrosation of glutathione (GSH) *in vivo*.^15^ In this system, the involvement of CytFe^2+^–NO^+^ was disproved by the authors. The proposed mechanism assumes that GSH binding to the cytochrome *c* occurs in the first reaction step, followed by a reaction with NO, leading to a CytFe^3+^–GS–N^•^–OH transition complex, which decomposes to reduced cytochrome *c* (CytFe^2+^) and GSNO.

It has also been reported that ferriprotoporphyrin IX can mediate GSNO formation by accepting an electron either from NO or GSH in the first reaction step.^17^ Under *in vitro* conditions, the feasibility of the two encountered reaction pathways (reaction of GSH with heme-Fe^2+^–NO^+^ and reaction of NO with heme-Fe^3+^–GS to form GSNO and ferroheme-NO) has been shown. However, only the latter was discussed as being more viable *in vivo*. For both mechanisms, the heme-Fe^2+^–RSNO complex has been postulated to be an intermediate.^16,17^ Formation of an iron-heme-coordinated *S*-nitrosothiol intermediate in the SNO^–^ formation pathway has been also proposed by Miljkovic et al.^23^ Despite the spectrophotometrically observed single-step transformation of [(Por)Fe^2+^–NO^+^] to [(Por)Fe^2+^–NO] in the presence of HS^–^, the application of high-resolution cryospray electrospray ionization time-of-flight (ESI-TOF) mass spectrometry allowed to confirm the formation of [(Por)Fe^2+^–NOS] as an intermediate preceding the formation of [(Por)Fe^2+^–NO].

Despite numerous studies devoted to *S*-nitrosothiols, the molecular mechanisms are still poorly understood and have multiple unresolved aspects. Due to the heterogeneity of the cellular environment and diversity of spatial and temporal patterns of NO generation and degradation, the variety of reaction pathways responsible for NO storage in a form of *S*-nitrosothiols is understandable. The biological significance of individual pathways generating *S*-nitrosothiols may change depending on, e.g., cell type, its compartment under consideration, its redox state, pathology, or aging.

In the presented studies, we have focused on the molecular aspects of *S*-nitrosothiol generation with the assistance of heme centers with one labile coordination site. Our goal was to verify if two reaction pathways, direct transfer of NO^+^ from the Fe^2+^–NO^+^ moiety to thiol and NO attack on the coordinated thiol (Fe^3+^–RS), can operate and be perceived as biologically relevant. The difficulties in the in-depth study of the heme-mediated generation of RSNO arise mainly from the elusiveness of heme-Fe^2+^–RSNO intermediates that upon detection would prove the actual reaction mechanism. Thus far, the kinetics of the heme-Fe^2+^–RSNO formation has not been addressed, which impedes the assessment of the biological relevance of this reactivity pathway.

To shed more light on this subject, we have performed a detailed quantitative and kinetic study on the formation of selected *S*-nitrosothiols using *N*-acetylmicroperoxidase-11 (AcMP-11) as an electron acceptor. AcMP-11 is a heme complex covalently attached to 11 amino acid residues obtained by proteolytic digestion of cytochrome *c*.^24^ The fifth coordination position in the heme is occupied by the imidazole group of His18, whereas in the sixth coordination position, a labile water molecule is present at neutral pH. The AcMP-11 reduction potential (*E*_Fe^2+^Fe^3+^_^°′^ = −134 mV vs SHE at pH 7) makes *S*-nitrosylation in the presence of AcMP-11 kinetically and thermodynamically feasible.^10^ The *S*-nitrosation was examined using three low-molecular-weight thiols: glutathione, cysteine, and *N*-acetylcysteine. Efficient *S*-nitrosation assisted by AcMP-11 has been confirmed in both reactivity scenarios, namely, NO reaction with pregenerated (AcMP-11)Fe^3+^(RS) and RS^–^ reaction with pregenerated (AcMP-11)Fe^2+^(NO^+^). The application of AcMP-11 as an electron acceptor allowed us to spectrophotometrically identify and kinetically characterize the formation of heme-Fe^2+^–RSNO species as an intermediate preceding the formation of ferrous-nitrosyl AcMP-11. Detailed analysis of the kinetic data answered an important question concerning ferri-heme-assisted molecular mechanism of NO storage as the low-molecular-weight *S*-nitrosothiols, providing unexpected evidence that only the nucleophilic attack of the RS^–^ group on (AcMP-11)Fe^2+^–NO^+^ is the productive reaction pathway, whereas the generation of (AcMP-11)Fe^3+^–RS is a dead-end equilibrium. The mode of reversible interaction of NO with proteins containing a transition-metal center is a central research topic.^25,26^ Herein, we report an in-depth kinetic characterization of the reversible capture of NO in the form of the (AcMP-11)Fe^2+^(RSNO) adduct. Presented mechanistic studies supported by theoretical calculations aim toward defining the molecular mechanisms of heme-mediated RSNO formation that would serve as a model for the analogous process assisted by heme proteins occurring in living cells.

## Materials and Methods

### Materials

All chemicals used in this study were of analytical reagent grade. Microperoxidase-11 (≥85%) sodium salt, l-glutathione reduced (GSH) (≥98%), *N*-acetyl-l-cysteine (AcCys) (≥99%), l-cysteine (Cys) 97%, sodium phosphate dibasic heptahydrate (≥99%), 2-3-diaminonaphthalene, sulfanilamide (≥98%), sodium nitrite (≥99%), *N*-ethylmaleimide (NEM) (≥98%), and diethylenetriaminepentaacetic acid (DTPA) (≥98%) were purchased from Sigma-Aldrich. NO gas (Linde UN 1660) was passed through the concentrated KOH solution to remove higher nitrogen species (NO_2_, N_2_O_3_) and subsequently through a column with Ascarite II (NaOH on silica gel, Sigma-Aldrich). All solutions were prepared in deionized water.

### Sample Preparation

Microperoxidase-11 was acetylated with acetic anhydride in carbonate buffer (0.2 M, pH = 9.3) at 4 °C according to the published procedure to prevent aggregation in an aqueous solution.^27^*N*-Acetylmicroperoxidase-11 (AcMP-11) was purified by dialysis using a Pur-A-Lyzer Mega Dialysis Kit, MWCO 1 kDa (Sigma-Aldrich) for 24 h. Subsequently, verification of peptide purity was carried out by the HPLC technique (Shimadzu LC 2030C), which confirmed the conversion of MP-11 to AcMP-11 to be close to 100%. A Brownlee Bio C18 Column (PerkinElmer, 5 μM, 150 mm × 4.6 mm) was employed for HPLC separation. 0.05% trifluoroacetic acid (TFA) in CH_3_CN (eluent A) and 0.05% TFA in H_2_O (eluent B) were used as the mobile phase with a flow rate of 1 mL min^–1^. Initially, the eluent ratio was 10:90 (A/B), and it was changed to 40:60 (A/B) within 20 min. All experiments with nitric oxide (NO) required anaerobic conditions. NO solution (1.7 mM) was prepared through the saturation of the deoxygenated phosphate buffer solution (pH = 7.4) with NO gas. The desired NO concentrations were obtained by dilution of the saturated solution with the deoxygenated phosphate buffer under an inert atmosphere.

*S*-Nitrosoglutathione (GSNO) and *S*-nitroso-*N*-acetylcysteine (AcCysNO) were synthesized according to the reported procedure.^28^ For this purpose, equimolar concentrations of GSH/AcCys and sodium nitrite were used. Next, hydrochloric acid was added to the reaction mixture, which was then vigorously mixed for 5 min in the dark. Subsequently, the solution was titrated with NaOH to obtain neutral pH. Samples were stored at −70 °C.

### AcMP-11-Mediated Formation of *S*-Nitrosothiols

The reactions of NO with thiols (GSH, Cys, and AcCys) in the presence of AcMP-11 were performed using a syringe-based mixing system under inert conditions. Solutions were prepared in phosphate buffer (0.025 M, pH 7.4) and supplemented with 0.25 mM DTPA in gas-tight glass syringes, followed by deoxygenation with argon. Quantitative determination of *S*-nitrosothiols was performed according to two procedures differing in the order in which the reagents were added to AcMP-11. The procedures were designed to compare the reactivity pathways occurring via (AcMP-11)Fe^3+^(RS) and (AcMP-11)Fe^2+^(NO^+^) intermediates on the *S*-nitrosothiol formation. All reactions were performed under inert conditions in the dark. In the first experimental approach, constant amounts of AcMP-11 (13.8 or 8.3 μM) were mixed with thiol (GSH, Cys, or AcCys) to generate (AcMP-11)Fe^3+^(RS), followed by the addition of properly concentrated solutions of NO. Two separate experiment types were performed following this order. One maintained the concentrations of thiols constant (1.38 mM) and varied the NO concentration (11.5–156 μM), while the other (performed for GSH only) varied the thiol concentration (0.05–1.8 mM) and maintained the NO concentration at 78 μM. In the second experimental approach, AcMP-11 was mixed with NO at first to generate (AcMP-11)Fe^2+^(NO^+^), and then varying concentrations of GSH (0.06–0.7 mM—final concentrations) were added. The final concentrations of AcMP-11 and NO were 13.8 μM and 78 or 39 μM, respectively.

In all reaction mixtures, the unreacted −SH groups were blocked by the addition of 6 mM of deoxygenated *N*-ethylmaleimide (NEM). The reaction mixture was allowed to react for 10 min in the dark. Then, the sample was aerated and 1 mM sulfanilamide was added to remove nitrite; the samples were allowed to react for 10 min in the dark. Quantification of *S*-nitrosothiols was performed immediately afterward with the spectrofluorimetric method and the electrochemical method with the application of the NO sensor.

### Spectrofluorimetric Analysis of *S*-Nitrosothiols

Concentrations of generated GSNO/AcCysNO/CysNO were determined using a fluorescent label (2,3-diaminonaphthalene—DAN).^29^ 170 μL of the reaction mixture was added to 3.83 mL of hydrochloric acid (0.25 mM) and supplemented with 400 μL of 0.05 mg/mL DAN in 0.62 M HCl. Each sample was divided into two equal parts. One was treated with 20 μL of HgCl_2_ (20 mM) to release NO from *S*-nitrosothiols, while to the second one (control sample), the same volume of water was added and allowed to react for 10 min in the dark. Then, 100 μL of NaOH (2.8 M) was added to all samples and allowed to incubate for 10 min before measuring the fluorescence spectra using an LS55 PerkinElmer spectrofluorimeter. Concentrations of *S*-nitrosothiols were determined from the fluorescence signal difference registered at 450 nm for the control sample and the sample treated with HgCl_2_. The fluorescence signals were converted to the actual amounts of the formed *S*-nitrosothiols using a GSNO calibration curve (Figure S1). Calibration solutions were prepared in an acid environment (0.25 mM HCl). Four milliliters of the samples containing 200–1100 nM GSNO was treated with 0.05 mg/mL DAN dissolved in 0.62 M HCl. Reaction mixtures were divided into two equal parts of 2.2 mL. Subsequently, one of them was treated with 20 μL of HgCl_2_ solution (0.17 mM), whereas the second half was treated with 20 μL of deionized water. Solutions were incubated for 10 min in the dark. Then, the samples were alkalized by treating with 100 μL of NaOH (2.8 M) and again left for 10 min. Fluorescence signals assigned to particular GSNO concentrations were the difference between the fluorescence registered at 450 nm for both standard samples. As a control, to check if the applied concentration of Hg^2+^ does not result in fluorescence quenching, the calibration curve with the application of freshly prepared NO_2_^–^ solutions was plotted (Figure S1). To check the accuracy of this procedure and exclude the influence of nitrite on the obtained results, we performed additional control experiments. First, an exact amount (7 μM) of GSNO was added to the solution containing 1 mM NO_2_^–^ and 1 mM GSH in Tris buffer pH 7.4, and the GSNO concentration was determined. The obtained value was 7.1 ± 0.4 μM, which corresponds well with the initial amount of GSNO. In the second experiment, GSNO generation was checked in a mixture of 13.8 μM AcMP-11, 0.6 mM GSH, and 0.1 mM NO_2_^–^. The control experiment did not reveal GSNO generation.

### Electrochemical Analysis of *S*-Nitrosothiols

The concentration of NO released from *S*-nitrosothiols was measured by an amiNO-700 sensor connected to a measuring system (Innovative Instruments, Tampa FL). The electrode was calibrated using GSNO and 1 mM Cu^2+^ supplemented with 5 μM ascorbic acid as a NO-releasing agent. Prior to the measurement, the electrode was immersed in 9.6 mL of Tris buffer (0.05 M, pH = 7.4, *T* = 25 °C) and then kept until signal stabilization. Subsequently, the buffer solution was supplemented with Cu^2+^ and ascorbic acid and incubated for 30 s before GSNO injection. A calibration curve was plotted as the dependence of the GSNO concentration (50–450 nM) vs the changes in the current signal. All experiments were performed in the dark. All experiments were carried out in a special flow glass vessel connected to a thermostat, which provided a constant temperature during experiments.

The quantitative determination of GSNO generated in the reaction of GSH with NO with the assistance of AcMP-11 was carried out in the same conditions as used for the calibration solutions. 200 μL of reaction mixture containing GSNO was injected into 9.6 mL of Tris buffer containing ascorbic acid (5 μM) and copper ions (1 mM), and the differences in the current signals were registered (Δ*I*). The Δ*I* was converted to the actual amount of *S*-nitrosothiol formed using a GSNO calibration curve (Figure S2A).

The electrochemical method was also used to follow GSNO formation in the presence of AcMP-11. All experiments were performed in phosphate buffer (0.025 M, pH = 7.4) under anaerobic conditions. After 10 min of bubbling with argon, nitric oxide was added to the buffer to achieve final concentrations equaling 400 or 550 nM. When the registered current reached a maximum, 50 μM GSH (control sample) or 1 μM AcMP-11 with 50 μM GSH was added. An argon atmosphere was kept over the solution during the whole experiment. Reaction mixtures were gently mixed until the electrochemical signal completely decayed. Then, 500 μM of Cu^2+^ solution was introduced to the mixture, and the changes in the current intensity were recorded. Illustrative confirmation of GSNO formation in the studied system is also reported in Figure S2B.

### Stopped-Flow Kinetics Measurements

Kinetics data for the reactions were recorded on an Applied Photophysics SX20 stopped-flow spectrometer equipped with a sequential mixing mode and a diode-array detector. Measurements were performed under a constant temperature provided by a thermostat unit (±0.1 °C) connected to the stopped-flow apparatus and under anaerobic conditions. All solutions were prepared in phosphate buffer (0.1 M, pH = 7.4) with the addition of 0.25 mM DTPA. In a typical experiment, deoxygenated AcMP-11 solution (5 × 10^–6^ M) was rapidly mixed with NO solution (1.8 × 10^–4^ M) in the first mixing drive. After a defined delay period, different concentrations of deoxygenated solutions of GSH, Cys, or AcCys (0.0005–0.003 M) were introduced to the system in a second mixing drive. The reactions were followed at 5 °C. Alternatively, deoxygenated AcMP-11 (5 × 10^–6^ M) was mixed with thiols (1.25 mM) in the first mixing drive. After a defined delay period, NO solutions (0.5–8.5 × 10^–4^ M) were introduced to the reaction mixture. These experiments were performed at 20 °C. All kinetic experiments were performed under pseudo-first-order conditions provided by using at least a 10-fold excess of NO or thiol (GSH, AcCys, or Cys) over AcMP-11. Absorbance changes were tracked at 413 nm, or time-resolved spectra were registered. Reported observed rate constants are mean values of at least six kinetic runs.

### UV–Vis Measurements

UV–vis spectral changes for the reaction of AcMP-11 with thiols were recorded on a Lambda 950 spectrometer (PerkinElmer) at 20 °C under anaerobic conditions.

### Theoretical Calculations

Two theoretical models of AcMP-11 were used for the study: a simplified model in which the heme moiety was reduced to Fe^3+^ porphin with imidazole as an axial ligand (referred to as model 1) and a full model consisting of the heme group, its side substituents, a part of the peptide backbone (Cys14-Ala15-Gln16-Cys17-His18), and explicit water molecules as developed previously^30^ (referred to as model 2)—see the Computational Analysis of *N*- and *S*-Iron-Heme-Coordinated *S*-Nitrosothiols section. The geometry and electronic properties of the possible complexes of AcMP-11 with NO, selected thiols (Cys, AcCys, GSH, and their simplified form modeled by the −SCH_3_^–^ group), and nitrosothiols (bound via different donor atoms—S and N) were elucidated.

The theoretical studies were performed using density functional theory (DFT), as implemented in Turbomole.^31^ The hybrid B3LYP functional^32,33^ was used with the def2-TZVP basis set^34^ for all of the atoms in model 1 and def2-SVP^34^ in model 2. The dispersion interactions were accounted for by applying Grimme +D3 correction.^35^ In the calculations, the solvent (water) was accounted for by applying the polarizable continuum model (COSMO)^36^ with permittivity ε = 80. The AcMP-11 thiol complexes were calculated as the low-spin systems following our calculations for (Por)Fe(SCH_3_)(Im), showing that structures of higher multiplicities had higher total energies: the energies of the intermediate-spin-state and high-spin-state structures are higher by 14.2 and 8.6 kcal/mol, respectively, relative to the low-spin structure. Similarly, the lowest possible multiplicity of the (Por)Fe(N(O)SCH_3_)(Im) complex was energetically favored (intermediate-spin-state and high-spin-state structures lie higher by 17.8 and 11.0 kcal/mol, respectively, to the low-spin complex). In the case of the (Por)Fe(S(NO)CH_3_)(Im) complex, the low-spin-state structure was the only one where the nitrosothiol stayed bound to the iron ion. The intermediate- and high-spin complexes were characterized by slightly lower total energies than the low-spin one (−0.6 and −1.3 kcal/mol), but the iron–sulfur distances exceeded 3.0 Å, indicating that there is no bonding between the metal center and the nitrosothiol.

## Results and Discussion

### Quantitative Analysis of *S*-Nitrosothiol Formation

**Scheme 1:**
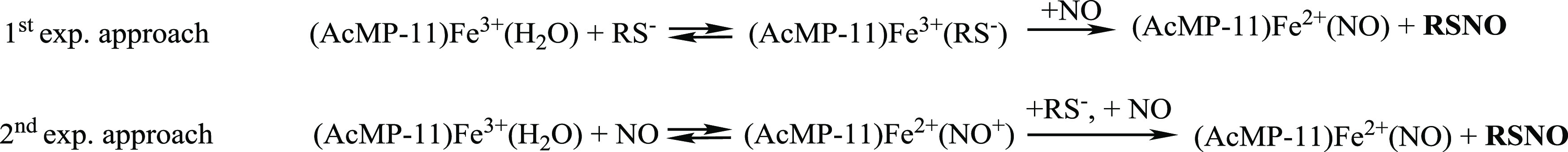
Two Reactivity Pathways for the Generation of RSNO

The ability of *N*-acetylmicroperoxidase-11 (AcMP-11) to mediate *S*-nitrosation was tested with the application of a fluorescence-based detection of *S*-nitrosothiols (RSNO). Three biologically relevant thiols (RS), glutathione, cysteine (Cys), and *N*-acetylcysteine (AcCys) were applied in the study. A literature survey revealed that it is commonly accepted that both porphyrin (Por) complexes, (Por)Fe^2+^(NO^+^) and (Por)Fe^3+^(RS), can be the source of RSNO via a nucleophilic attack of thiol or NO, respectively.^17,26,37^ Therefore, two reactivity pathways were tested for the capacity of RSNO generation (Scheme 1) either through combining (AcMP-11)Fe^3+^(RS) with NO or by mixing pregenerated (AcMP-11)Fe^2+^(NO^+^) with the thiol. Due to the relatively low AcMP-11 redox potential, none of the applied thiols caused the reduction of (AcMP-11)Fe^3+^ under the studied conditions.^38,39^

**Figure 1:**
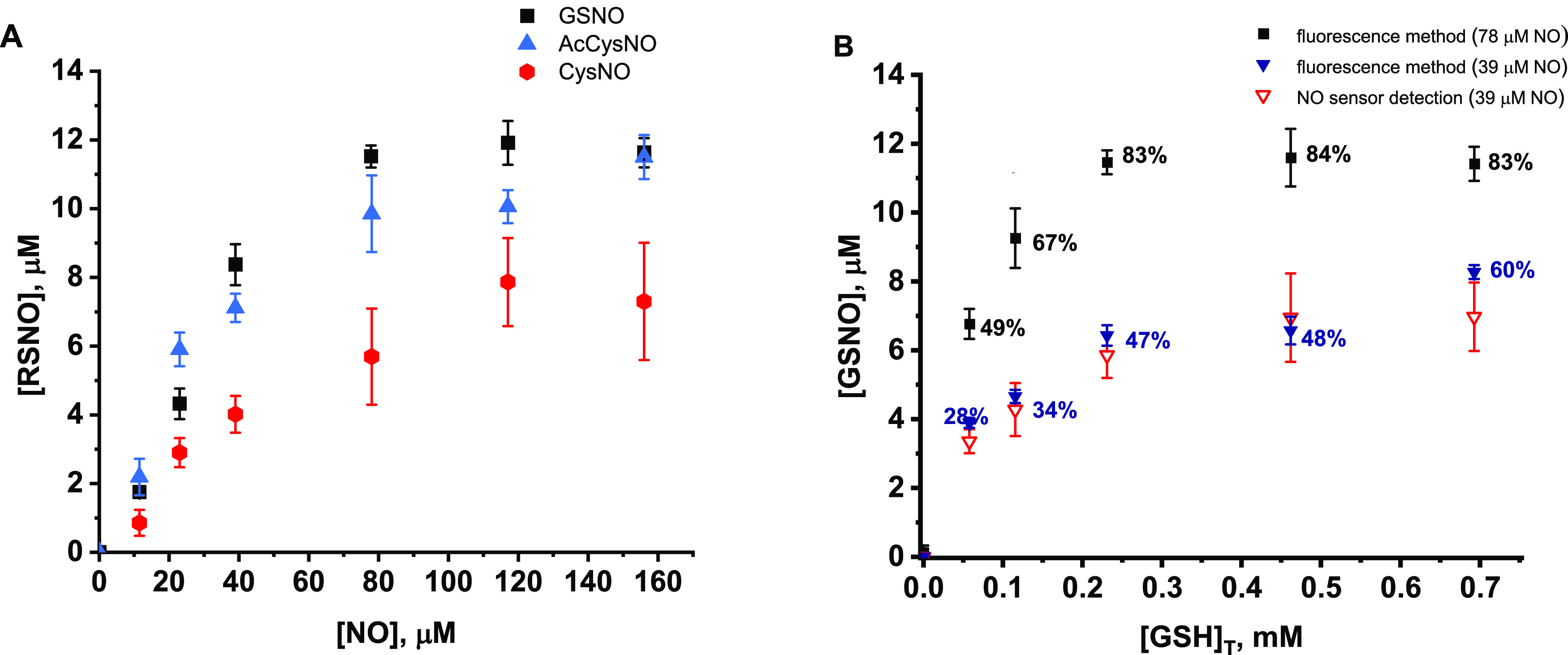
Quantitative analysis of RSNO formation. (A) NO concentration-dependent formation of RSNO obtained by the reaction of (AcMP-11)Fe^3+^(RS) with NO in the presence of 1.38 mM [RS]_T_. (B) GSH concentration-dependent formation of GSNO obtained by the reaction of (AcMP-11)Fe^2+^(NO^+^) with GSH in the presence of constant NO concentrations 78 μM (black squares) and 39 μM analyzed with two methods: fluorescence (blue full triangles) and NO-selective sensor (red empty triangles). The values expressed in % are the yields of [GSNO] calculated in terms of [AcMP-11]. Experimental conditions: [MP-11] =13.8 μM, [phosphate buffer] = 0.025 M, pH = 7.4, [DTPA] = 0.25 mM, *T* = 22 °C; electrochemical analysis [Tris buffer] = 0.05 M and pH = 7.4; [Cu^2+^] = 1 mM; [ascorbic acid] = 5 μM. [RS]_T_ is the total thiol concentration.

Nitric oxide transfer to GSH was tested by applying both experimental approaches, whereas comparative experiments with the application of Cys and AcCys were performed only with the first approach. The reactions were carried out at a physiological pH value (7.4) and under anaerobic conditions to keep the defined NO concentration and prevent oxygen-dependent *S*-nitrosation. Since the role of nitrite in RSNO generation has been shown previously,^40^ the concentration of nitrite in saturated NO solution was determined (∼0.15 mM) and its influence on the observed RSNO generation and fluorescence assay was excluded (for details, see Spectrofluorometric Analysis of S-Nitrosothiols). Efficient AcMP-11-mediated formation of RSNO products was confirmed in the presence of NO and RS excess over AcMP-11 irrespective of the reactant mixing order, whereas application of equimolar concentrations of NO, GSH, and AcMP-11 results in a relatively low GSNO generation (7.8 ± 0.8% calculated on [AcMP-11]). For all three thiols (1.38 mM), the amount of generated RSNO was studied as a function of NO concentration in the presence of 13.8 μM AcMP-11 (first experimental approach, see Scheme 1). In all cases, a similar saturated character of the dependence of the amount of generated RSNO vs [NO] was observed (Figure 1A). About 80 μM NO (approximately 6-fold the excess of NO over AcMP-11) was required to achieve the maximum conversion of GSH to GSNO. For all three thiols, the highest amounts of *S*-nitrosothiol did not exceed the AcMP-11 concentration, reaching a maximum of approx. 86% for GSNO, 83% for *N*-acetyl-*S*-nitrosocysteine (AcCysNO), and 57% for *S*-nitrosocysteine (CysNO) calculated in terms of [AcMP-11]. When analyzing the data in terms of NO concentration, the highest NO conversion to RSNO achieved was about 20% for GSH and AcCys, whereas for Cys, the detected amounts were about 2 times lower.

Lowering the concentration of AcMP-11 to 8.3 μM diminishes the amount of generated GSNO, whereas excluding AcMP-11 from the reaction mixture resulted in negligible GSNO generation (0.5 ± 0.3 vs 8.4 ± 0.6 μM determined in the presence of 13.8 μM AcMP-11), indicating that the AcMP-11 concentration is a parameter that governs the amount of *S*-nitrosothiol formation in the presence of appropriate high excesses of NO or thiol (Figure S3A).

GSNO formation was also studied in a function of [GSH]_T_ in the presence of a constant [NO] = 78 μM. Again, it was observed that GSNO generation is a saturable phenomenon and depends on [GSH]_T_ only at lower concentrations (up to approx. 0.5 mM) (Figure S3B). The highest conversion of NO to GSNO can be achieved using an excess of approximately 36 times of [GSH]_T_ over [AcMP-11].

The formation of GSNO was also analyzed in the second experimental approach according to Scheme 1. Experiments were performed for a constant [NO] of either 78 or 39 μM in a function of [GSH]_T_. The results reported in Figure 1B indicate that, also in this case, the formation of GSNO depends on both NO and GSH concentrations. The highest achieved [GSNO] corresponds well with the amount of GSNO determined for the same NO concentrations in the experiment performed under the first experimental approach (compare Figure 1A,B), showing that, also in this case, the NO concentration is one of the factors limiting the yield of RSNO. To verify the repeatability of the results obtained with the fluorescence method, samples obtained for 39 μM NO were additionally analyzed electrochemically (an alternative RSNO detection method) with the application of a NO sensor (Figure 1B compares the dependencies represented by the red and blue triangles). The consistency of the results from the electrochemical and fluorescence method confirms the reliability of the determined GSNO concentrations.

### UV–Vis Spectral Study

**Figure 2:**
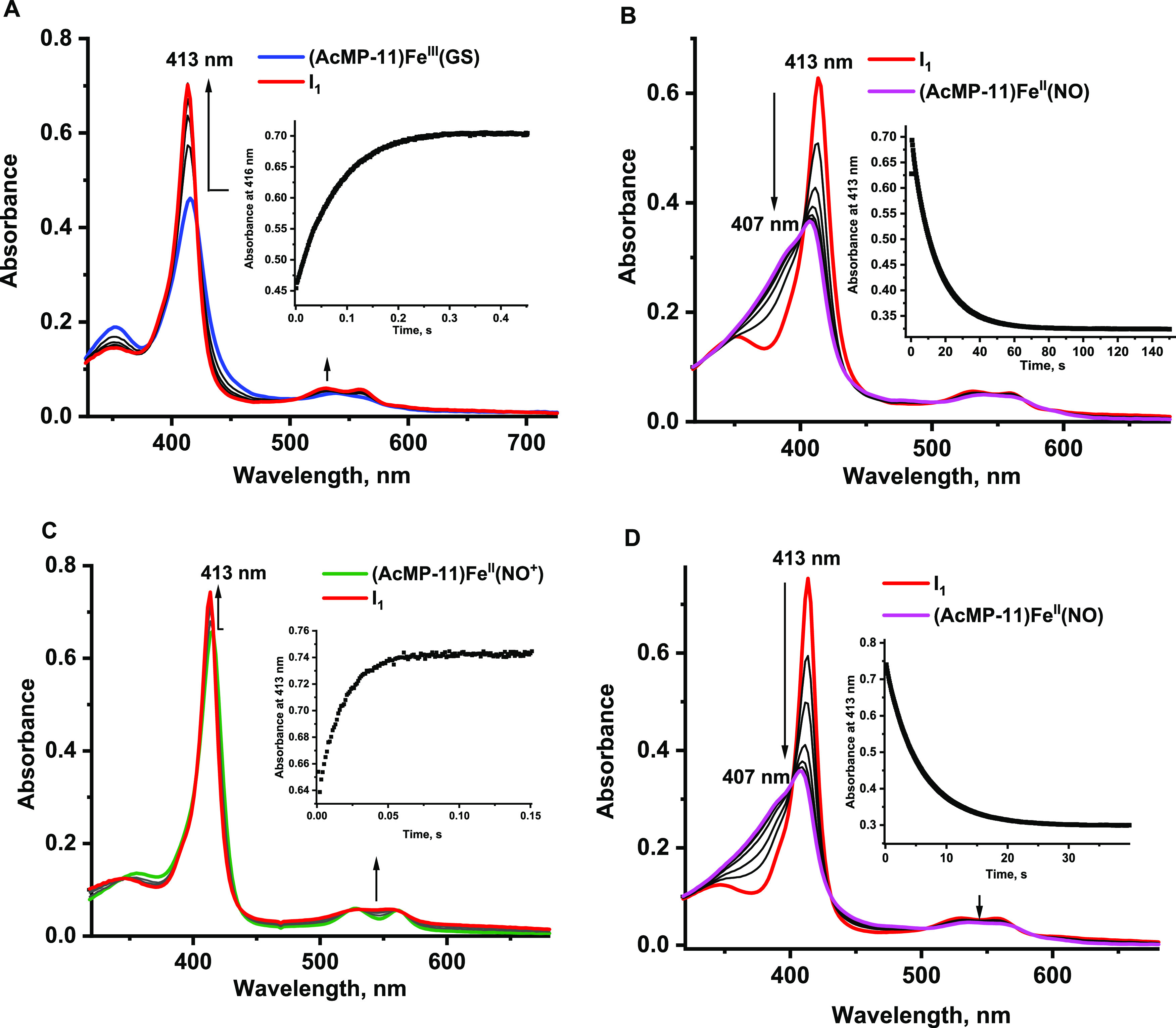
(A) Representative spectra for the formation of **I**_**1**_ (red line) in the reaction of (AcMP-11)Fe^3+^(GS) (blue line) with NO; the inset presents a kinetic trace at 413 nm, (B) transformation of **I**_**1**_ to (AcMP-11)Fe^2+^(NO) registered in the second reaction step. (C) Representative spectra for the formation of **I**_**1**_ (red line) in the reaction of (AcMP-11)Fe^2+^(NO^+^) (green line) with AcCys; the inset presents a kinetic trace at 413 nm, (D) transformation of **I**_**1**_ to (AcMP-11)Fe^2+^(NO) registered in the second reaction step. Experimental conditions: [AcMP-11] = 5 × 10^–6^ M, [GSH]_T_ = 0.002 M, [AcCys]_T_ = 0.003 M, [NO] =1.8 × 10^–4^ M, [phosphate buffer] = 0.1 M, [DTPA] = 0.25 mM, pH = 7.4, *T* = 5 °C.

The reaction pathways introduced in Scheme 1 were analyzed spectrophotometrically by following UV–vis electronic spectra of AcMP-11 with a stopped-flow premix system (pH = 7.4, anaerobic conditions). At pH 7.4, AcMP-11 exists as a six-coordinate complex with coordinated His and labile H_2_O ligands in the axial positions.^24^ Depending on the experimental approach, either (AcMP-11)Fe^3+^(RS) (first approach) or (AcMP-11)Fe^2+^(NO^+^) (second approach) was initially generated in the first mixing drive by the substitution of labile H_2_O ligands with RS or NO, respectively. Figure 2A illustrates the time-resolved spectral changes after the addition of NO to (AcMP-11)Fe^3+^(GS). The initial spectrum (blue) displays a low-intensity Soret band at 415 nm and a Q band at 536 nm characteristic for (AcMP-11)Fe^3+^(RS) complexes.^38^ Addition of NO resulted in a blue shift in the Soret band to 413 nm and an increase in the intensity of the Q bands (red spectrum). This transient intermediate (**I**_**1**_) was unstable and further underwent a much slower transformation expressed through the Soret band collapse and shift to 407 nm, characteristic of a ferrous-nitrosyl complex of microperoxidase—(AcMP-11)Fe^2+^(NO). The complex possessing the same spectra can be obtained by the addition of NO to the ferrous-AcMP-11 (Figure S4) and also as a product of reductive nitrosylation occurring after NO binding by ferric-AcMP-11 under basic conditions.^30^ For clarity, the representative transformation of **I**_**1**_ to (AcMP-11)Fe^2+^(NO) was illustrated separately in Figure 2B. When the analogous reactions were carried out with (AcMP-11)Fe^3+^(AcCys) or (AcMP-11)Fe^3+^(Cys), similar spectral changes were registered.

Global spectral analysis of the reaction course according to the second experimental approach revealed that here a double-step process is observed but the transformation to **I**_**1**_ is much faster in this case. Figure 2C illustrates the representative transformation of (AcMP-11)Fe^2+^(NO^+^) after mixing with AcCys to the transient intermediate. The initial spectrum of (AcMP-11)Fe^2+^(NO^+^) (green—λ_max_ = 415, 527, and 561 nm)^30^ was slightly blue-shifted, indicating the formation of an intermediate with UV–vis spectral features (λ_max_ 413 nm and broad Q band at 522–566 nm, Figure 2C red spectrum) analogous to the ones registered for I_1_ in the reaction of (AcMP-11)Fe^3+^(RS) with NO (compare Figure 2A,C). Also in this case, the transient intermediate was converted to a ferrous-nitrosyl complex (Figure 2D). The addition of Cys or GSH resulted in similar spectral changes. The spectra analysis indicates that the same intermediate (**I**_**1**_) is generated in both experimental approaches. An assumption can be made that **I**_**1**_ would be the complex with coordinated *S*-nitrosothiol (AcMP-11)Fe^2+^(RSNO), which in the consecutive reaction transforms to the ferrous-nitrosyl complex.

Although the generation of *S*-nitrosothiols in the presence of ferric-heme electron acceptors has already been reported, the intermediate that mediates the reaction has not yet been spectrophotometrically registered. Unfortunately, the instability of (AcMP-11)Fe^2+^(RSNO) originating from the relatively fast transformation to (AcMP-11)Fe^2+^(NO) presents a substantial challenge for its isolation and unambiguous characterization.

**Scheme 2:**
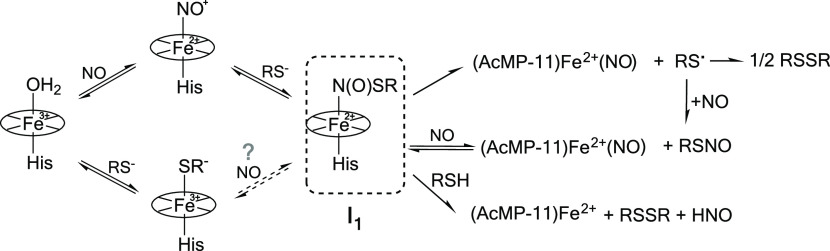
Schematic Representation of the Observed Formation of **I**_**1**_ and Its possible Decomposition Pathways

Since, regardless of the reactivity scenario (first or second experimental approach), the spectra of the captured intermediate products are identical, the same coordination mode of *S*-nitrosothiol to iron is most likely present in both cases. This is quite surprising because the nucleophilic attack of NO on the coordinated thiol group in (AcMP-11)Fe^3+^(RS) should result in the formation of *S*-coordinated RSNO in (AcMP-11)Fe^2+^–S(R)NO, which would be expected to exhibit a different spectral characteristic from *N*-coordinated RSNO in (AcMP-11)Fe^2+^–N(O)SR. Based on the spectrum of **I**_**1**_, the results of the kinetic studies, and the theoretical calculations presented in the next chapters, we infer that the observed intermediate is an *N*-coordinated *S*-nitrosothiol complex (AcMP-11)Fe^2+^–N(O)SR. Our conclusion stays in agreement with the previously observed preferred RSNO binding mode *via* the N-atom in the transition-metal-assisted NO capture in a metal-coordinated *S*-nitrosothiol adduct.^25,26,41,42^ Schematic representation of the observed formation of **I**_**1**_ and its possible decomposition pathways (RSH reaction with coordinated RSNO resulting in RSSR and HNO formation, homolytic S–N bond splitting leading to RS^•^ release, and substitution of coordinated *S*-nitrosothiol by NO with the release of free RSNO) is presented in Scheme 2 (the scheme is meant to be illustrative and does not depict the complete reaction scheme).

Ferri-heme-mediated generation of *S*-nitrosothiols under inert conditions leads to the reduction of the iron center to Fe^2+^, which is trapped in the form of (AcMP-11)Fe^2+^(NO). The formation of the inert ferrous-nitrosyl complex not only shuts down the decomposition of generated *S*-nitrosothiol^26^ but also limits the reformation of the ferric state of the AcMP-11 center. Catalytic generation of *S*-nitrosothiols would require oxidation of the Fe^2+^–NO center to the ferric one. The ability of (AcMP-11)Fe^2+^(NO) to regain the activity toward *S*-nitrosothiol formation by O_2_ oxidation was verified, showing that very low O_2_ concentration converts the ferrous-nitrosyl complex to (AcMP-11)Fe^3+^(RS), which after the addition of a fresh portion of NO regenerated (AcMP-11)Fe^2+^(NO) with an observable **I**_1_ intermediate. This procedure could be repeated several times.

### Kinetic Studies

We took advantage of the fact that we successfully registered the unstable intermediate (**I**_**1**_) and performed detailed kinetic studies on its generation in both reactivity pathways (Scheme 1). The reaction kinetics were followed, registering the absorbance changes at 413 nm under pseudo-first-order conditions.

#### Formation of **I_1_** in the Reaction of (AcMP-11)Fe^3+^(RS) with NO

In the case of the first reactivity scenario, the reaction was followed in a function of NO concentration in the presence of 3 mM [RS]_T_, providing a satisfactory conversion of (AcMP-11)Fe^3+^(H_2_O) to (AcMP-11)Fe^3+^(RS) (>97% depending on RS). The thiol excess was chosen based on the apparent binding constants (*K*_app_^RS,th^) of RS to AcMP-11 (Table S1). The *K*_app_^RS,th^ values were calculated for each thiol from the spectral changes (Figure S5) registered for the reaction shown in eq 2.
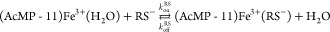
2

The kinetic studies on the reaction shown in eq 3 allowed determining the observed rate constants (*k*_obs_^1^) from the single-exponential kinetic traces registered for the first reaction step, the formation of the **I**_**1**_ intermediate (Figure 2A—insets).
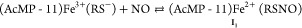
3

**Figure 3:**
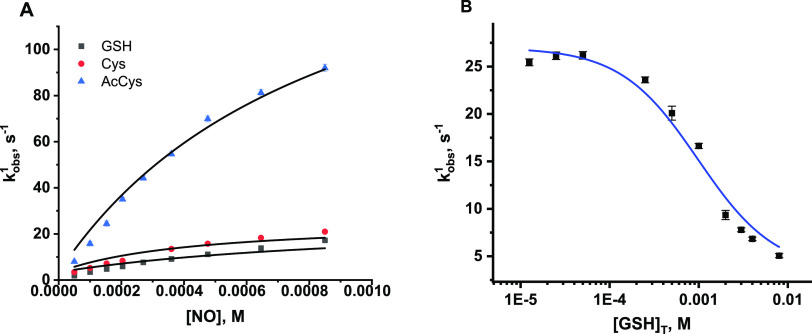
Dependencies of ***k***_**obs**_^**1**^ (A) in a function of NO concentration in the presence of 3 mM [RS]_T_ and (B) in a function of [GSH]_T_ in the presence of 0.36 mM NO. The solid lines represent the results of simulations obtained using rate constants reported in Table S1 and obtained previously (***k***_**on**_^**NO**^ = 3.7 × 10^6^ M^–1^ s^–1^, ***k***_**off**_^**NO**^ = 3.4 s^–1^ at 20 C°).^30^ Experimental conditions: [AcMP-11] = 5 × 10^–6^ M, [phosphate buffer] = 0.1 M, [DTPA] = 0.25 mM, pH = 7.4, *T* = 20 °C.

**Scheme 3:**
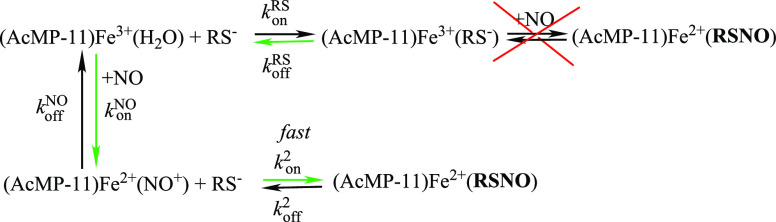
Reactivity Pathway for RSNO Formation after NO Addition to (AcMP-11)Fe^3+^(RS^–^) The green arrows indicate the reaction route.

As seen in Figure 3A, the dependence of *k*_obs_^1^ on [NO] is not linear and appears to have a saturation character with increasing NO concentration. Meticulous data analysis indicated that such behavior correlates with the mechanism where (AcMP-11)Fe^3+^(RS) is inactive in the generation of RSNO, meaning that eq 2 constitutes a dead-end equilibrium. Therefore, the formation of **I**_**1**_ observed in the first reactivity pathway has to occur by thiol binding to the Fe^2+^–NO^+^ moiety preceded by the reaction of NO with (AcMP-11)Fe^3+^(H_2_O) staying in equilibrium with the (AcMP-11)Fe^3+^(RS) form. The green arrows in Scheme 3 (the scheme is meant to be illustrative and does not depict the complete reaction) indicate the proposed reactivity pathway for RSNO formation. According to the proposed mechanism, at appropriately high NO concentration (unfortunately unreachable under aqueous conditions) *k*_obs_^1^ should be independent of [NO] and reach values corresponding to *k*_off_^RS^ for each thiol. Such behavior can be accounted for in terms of a limiting dissociative mechanism, in which the release of thiol from (AcMP-11)Fe^3+^(RS) is the rate-determining step in the formation of the **I**_**1**_ complex at high NO concentrations.

Taking into account that the rapid attack of RS^–^ nucleophile on the Fe^2+^–NO^+^ center (see Formation of **I1** in the Reaction of (AcMP-11)Fe2+(NO+) with RS) is limited by the formation of (AcMP-11)Fe^3+^(H_2_O) from (AcMP-11)Fe^3+^(RS), followed by nitrosylation of the aqua complex (Scheme 3), the rate of *k*_obs_^1^ can be expressed by eq 4.
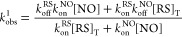
4

Under the conditions of high [NO], it can be assumed that *k*_on_^RS^[RS]_T_ ≪ *k*_on_^NO^[NO] and *k*_on_^RS^*k*_off_^NO^[RS]_T_ ≪ *k*_off_^RS^*k*_on_^NO^[NO]. Consequently, the rate law (eq 4) can be simplified to the following expression: *k*_obs_^1^ ≅ *k*_off_^RS^, which is in agreement with the observed curvature in the dependencies shown in Figure 3A. Taking into account too many variables in eq 4 and the inability to achieve the NO concentration, at which the dependence reported in Figure 3A completely plateaus, the credibility of the proposed mechanism was confirmed by simulations instead of direct data fitting. Simulation of the dependence expressed by eq 4 was performed using the applied [RS]_T_ concentration, the determined rate constants for RS binding (Figure S6) to (AcMP-11)Fe^3+^(H_2_O) reported in Table S1, and the rate constants for (AcMP-11)Fe^2+^(NO^+^) formation reported previously.^30^ Simulated dependencies display satisfactory agreement with the course of the points obtained experimentally, indicating that the formation of **I**_**1**_ in the first experimental approach depends on the rate constants governing the formation of a reactive (AcMP-11)Fe^2+^(NO^+^) complex from (AcMP-11)Fe^3+^(RS) under applied conditions. To support the proposed reaction mechanism, additional studies were performed, in which the NO concentration was maintained constant, whereas the GSH concentration was varied. The applied wide range of GSH concentration allowed recreating the sigmoidal-shaped dependence of *k*_obs_^1^ vs [GSH]_T_ expressed by eq 4 (Figure 3B). It can be observed that, under low GSH concentrations, *k*_obs_^1^ reaches a constant value which is in agreement with the fact that, under low [GSH]_T_, eq 4 can be reduced to *k*_obs_^1^ ≅ *k*_off_^RS^. For higher [GSH]_T_, a decrease in the *k*_obs_^1^ with increasing [GSH]_T_ and subsequent curvature in the dependence was observed, indicating reaching the conditions in which *k*_obs_^1^ ≅ *k*_off_^NO^. Such behavior confirms the proposed reaction mechanism, the credibility of which was supported by the simulation of eq 4, represented by the blue line in Figure 3B, with the applied NO concentration, kinetic rate constants for RS, and NO binding to (AcMP-11)Fe^3+^(H_2_O).

Reported data excludes the nucleophilic attack of NO on Fe^3+^–RS moieties as a source of RSNO generation in AcMP-11-assisted reactions. The kinetic analysis strongly supports that the formation of RSNO, quantitatively confirmed in the first experimental pathway, is consistent with the reaction pattern shown in Scheme 3, whereby NO transfer to the thiol occurs from the (AcMP-11)Fe^2+^(NO^+^) reactive species. This result stays in agreement with the formation of N-atom-coordinated *S*-nitrosothiol in **I**_**1**_ (Scheme 2), namely, (AcMP-11)Fe^2+^-N(O)SR. Recently Poptic and Zhang^26^ pointed out that the decomposition of *S*-nitrosothiols on iron complexes is facilitated by *S*-atom coordination to Fe^2+^, whereas *N*-coordination strengthens the S–N bond in Fe^2+^N(O)SR species. Their conclusion complements our mechanism of RSNO formation excluding the S-atom coordination and supporting N-atom coordination of RSNO to the iron center of AcMP-11 in **I**_**1**_ before the transformation to (AcMP-11)Fe^2+^(NO).

In the literature, one can find spectroscopic and crystallographic data supporting the heme-assisted *S*-nitrosation of proximal thiolate in proteins and synthetic heme-thiolate complexes.^16,43^ However, it is worth stressing that the reported cases concern the proximal Cys residue from a polypeptide chain^16^ or a thiolate covalently linked to the porphyrin^43^ (not a free low-molecular-weight thiol), and importantly, the *S*-nitrosation step was preceded by the formation of NO^+^–Fe^2+^–SR intermediate. The nature of the ligand coordinated in the trans-position to the thiol (in AcMP-11, trans-position is blocked by His) may be relevant to the feasibility of its *S*-nitrosation.

#### Formation of **I_1_** in the Reaction of (AcMP-11)Fe^2+^(NO^+^) with RS

**Figure 4:**
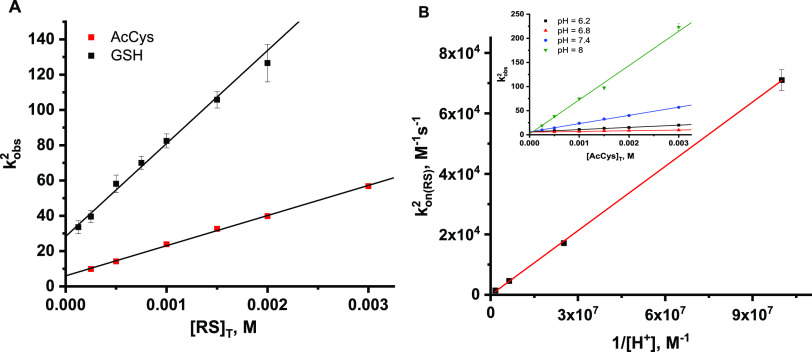
(A) Dependencies of *k*_obs_^2^ in a function of [RS]_T_ concentration; RS—glutathione (black squares) or *N*-acetylcysteine (red squares). (B) Dependencies of *k*_on(AcCys)_^2^ in a function of 1/[H^+^] for the reaction of (AcMP-11)Fe^2+^(NO^+^) with AcCys, the inset presents dependencies of *k*_obs^2^_ vs [AcCys]_T_ at selected pH values. Experimental conditions: [AcMP-11] = 5 × 10^–6^ M, [NO] = 1.8 × 10^–4^ M, [phosphate buffer] = 0.1 M, [DTPA] = 0.25 mM, *T* = 5 °C.

**Figure 5:**
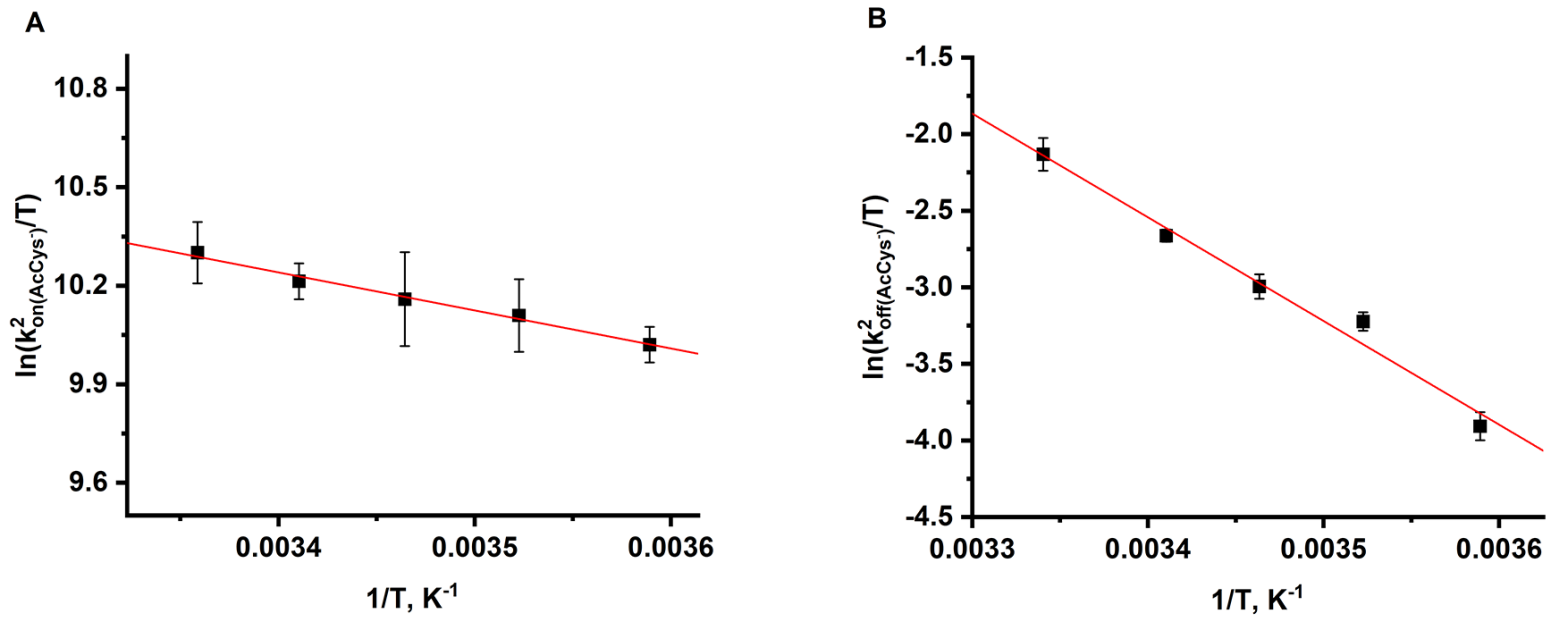
Eyring plots for the forward (A) and back (B) reactions of (AcMP-11)Fe^2+^(N(O)AcCys) formation in the reaction of (AcMP-11)Fe^2+^(NO^+^) with AcCys. Experimental conditions: [AcMP-11] = 5 × 10^–6^ M, [phosphate buffer] = 0.1 M, [NO] = 1.8 × 10^–4^ M, and [DTPA] = 0.25 mM.

Stopped-flow kinetic studies on the second reactivity scenario were performed in the presence of NO excess being a source of (AcMP-11)Fe^2+^(NO^+^) that was mixed with [RS]_T_ (eq 5). In this reactivity scenario, **I**_**1**_ formation was much faster than in the first approach and required the kinetic experiments to be carried out at 5 °C. Unfortunately, the reaction with Cys was still too fast to be quantified even at 5 °C, which allowed us to obtain kinetic data only for the reactions with GSH and AcCys. The *k*_obs_^2^ values determined from single-exponential kinetic traces (Figure 2C) show a linear dependence on the thiol concentration with a significant intercept (Figure 4A), indicating the reversibility of the process and thus can be expressed by eq 6.
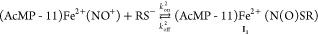
5
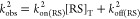
6Linear function fitting allowed determining the apparent *k*_on(RS)_^2^ and *k*_off(RS)_^2^ values from the slope and intercept, respectively (Figure 4A, Table 1). Based on the previous studies of the nucleophilic thiol attack on the transition-metal-nitrosyl complexes, it is expected that, in reaction 5, the deprotonated forms of the thiols (RS^–^) could be the sole reactive species.^44,45^ To verify this, *k*_obs_^2^ values for AcCys binding (as a representative thiol) were measured at the pH range of 6.2–8.0 (Table 1). Considering the acid–base equilibrium of thiols (reaction 7), we arrive at the rate constants for the forward and back reactions expressed by eqs 8 and 9.
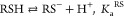
7
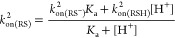
8
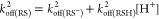
9Under conditions [H^+^] ≫ *K*_a_eq 8 can be simplified to
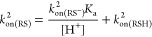
10and then the dependence of *k*_on(RS)_^2^ vs 1/[H^+^] allows determining the *k*_on(RS^–^)_^2^ value from the slope and *k*_on(RSH)_^2^ from the intercept of the linear function fitting (Figure 4B). Analysis of the results reported in Figure 4B revealed a linear distribution of the data with a lack of an intercept, indicating that *k*_on(RSH)_^2^ is zero (with a relatively large statistical error bar). This confirms the assumption that RS^–^ is the only reactive species and the apparent rate constants (*k*_on(RS)_^2^) cannot be compared due to the different p*K*_a_ values of the studied thiols. To calculate *k*_on(AcCys^–^)_^2^, the p*K*_a_ = 9.95 at *T* = 5 °C for the −SH group needed to be determined (for details, see the comment in Supporting Information Figure S7 and Table S2). Following the results obtained for AcCys, it was assumed that only the deprotonated form of GSH is reactive and *k*_on(GS^–^)_^2^ was calculated by extrapolating the data from eq 10 (Table 1). Analysis of the rate constants for the back reaction determined for AcCys at various pH values indicates that *k*_off(RSH)_^2^ is also negligible, which results in *k*_off(RS^–^)_^2^ = *k*_off(RS)_^2^. Figure 5 shows Eyring plots for the forward and back reactions of AcCys^–^ binding to (AcMP-11)Fe^2+^(NO^+^) obtained by examining the effect of temperature on *k*_obs(AcCys)_^2^ (Figure S8). The temperature-dependent kinetic studies were performed only for AcCys since the observed rate constants for the formation of **I**_**1**_ were low enough to be followed at higher temperatures using a stopped-flow apparatus. To calculate  from obtained  values, we determined the  values at desired temperatures (Table S2), whereas for the back reaction, the assumption, based on the previous conclusions that *k*_off(AcCys^–^)_^2^ = *k*_off(AcCys)_^2^, has been made. Linear correlation of  or  vs 1/*T* (Figure 5) allowed determining the activation enthalpy (Δ*H*^‡^) and entropy (Δ*S*^‡^) from the slope and intercept, respectively. The resulting rate constants and associated activation parameters for the reversible formation of (AcMP-11)Fe^2+^(N(O)AcCys) are reported in Table 1.

**Table 1:** Kinetic Data for the Formation of (AcMP-11)Fe^2+^(N(O)SR) in the Reaction of AcCys and GSH with (AcMP-11)Fe^2+^(NO^+^)^a^

		AcCys/(GSH)		
*T*, °C	pH	*k*_on(RS)_^2^, M^–1^ s^–1^ × 10^–4^	*k*_on(RS^–^)_^2^, M^–1^ s^–1^ × 10^–6^^b^	*k*_off(RS^–^)_^2^, s^–1^
5	6.3	0.14 ± 0.01	6.32 ± 0.06^c^*/*(4.3 ± 0.2)^d^	5.4 ± 0.2
5	6.8	0.46 ± 0.01		6.1 ± 0.2
5	7.4	1.76 ± 0.05/(5.3 ± 0.2)		5.9 ± 0.4/(28 ± 2)
5	8.0	7.1 ± 0.3		1.5 ± 1.9
10	7.4	2.3 ± 0.1	7.0 ± 0.3^d^	11.3 ± 0.6
15	7.4	3.0 ± 0.1	7.4 ± 1.0^d^	14.4 ± 0.9
20	7.4	3.6 ± 0.1	8.0 ± 0.4^d^	20.4 ± 0.8
25	7.4	4.9 ± 0.4	8.9 ± 0.8^d^	35.5 ± 3.8
Δ*H*^‡^, kJ/mol			9.4 ± 0.5	56 ± 4
Δ*S*^‡^, J/mol·K			–80 ± 2	–27 ± 15
Δ*G*^‡^ (5 °C), kJ/mol			32 ± 1	63 ± 8

a For (AcMP-11)Fe^2+^(N(O)GS), only selected values were determined (reported after the slash).

b Calculated using p*K*_a_ determined in this study (SI, Table S2): p*K*_a_^AcCys, 5 ^°^C^ = 9.95; p*K*_a_^GSH, 5 ^°^C^ = 9.32.

c Determined from the dependence illustrated in Figure 4B.

d Estimated from eq 10 assuming *k*_on (RSH)_^2^ equals 0.

The *k*_on(AcCys^–^)_^2^ values determined in the present study are not influenced by temperature in a significant way. Obtained data indicate a relatively small activation barrier for the formation of **I**_**1**_ and a significantly negative Δ*S*_on_^‡^. Second-order rate constants lower than the diffusion rate by several orders of magnitude, together with the negative Δ*S*_on_^‡^, indicate that the N(O)–SR bond formation is the rate-determining step. On the other hand, the bond formation between coordinated NO^+^ and RS^–^ is expected to be accompanied by charge neutralization, which results in a decrease in electrostriction, accounting for the positive activation entropy contribution. Therefore, the negative Δ*S*_on_^‡^ suggests that the bond formation and charge concentration contributions prevail in the activation entropy for **I**_1_ formation. Taking into account the overall negative charge of AcMP-11, which results in the reduction of the electrophilicity of coordinated NO^+^, the obtained results stay in line with the higher contribution of bond formation, compared to decreasing electrostriction, in the nucleophile attack on coordinated NO^+^ in porphyrins with negatively charged substituents.^46,47^ According to the principle of microscopic reversibility, the “off” reaction should be dominated by N(O)–SR bond breaking, which should have a positive contribution to the Δ*S*_off_^‡^. The negative Δ*S*_off_^‡^ determined for the back reaction has to reflect the significant increase in solvent electrostriction related to bond braking and charge formation on the Fe^2+^(NO^+^) moiety. In this case, the increase in electrostriction compensates the intrinsic entropy contribution expected for the bond breaking.

### Thermodynamic Parameters for the Formation of I_1_ in the Reaction of RS^–^ with (AcMP-11)Fe^2+^(NO^+^)

**Figure 6:**
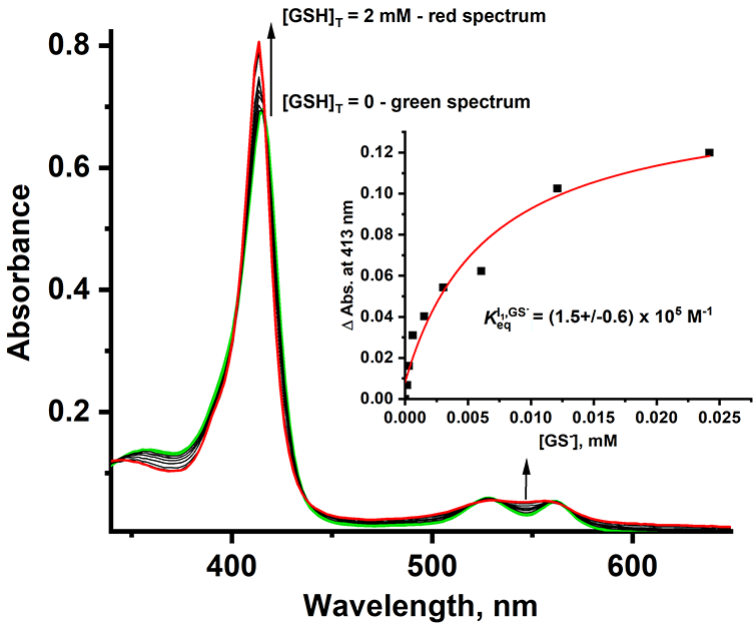
UV–vis spectra of (AcMP-11)Fe^2+^(GSNO) staying in equilibrium with (AcMP-11)Fe^2+^(NO^+^) obtained from the time-resolved spectral changes registered in the presence of various GSH concentrations. Inset illustrates the change in absorbance at 413 nm for the reaction of (AcMP-11)Fe^2+^(NO^+^) with glutathione. To calculate ***K***_**eq**_^**I**_**1**_,**GS-**^, the concentration of GS^–^ was used. Experimental conditions: [MP-11] = 5 × 10^–6^ M, [GSH]_T_ = (0–0.002) M, [NO] = 1.8 × 10^–4^ M, [phosphate buffer] = 0.1 M, [DTPA] = 0.25 mM, pH = 7.4, and T = 5 °C.

Due to the much slower transformation of coordinated *S*-nitrosothiol to ferrous-nitrosyl species compared to **I**_**1**_ generation, it was feasible to extract UV–vis spectra of **I**_**1**_ corresponding to the state of equilibrium and estimate equilibrium constants. Figure 6 presents spectra of **I**_**1**_ staying in equilibrium with (AcMP-11)Fe^2+^(NO^+^) registered in the presence of various GSH concentrations (analogous spectra in a function of Cys and AcCys are presented in Figure S9).

Data analysis revealed that the complete conversion of (AcMP-11)Fe^2+^(NO^+^) to the **I**_**1**_ complex requires approx. a 400-fold excess of thiol over AcMP-11 at pH 7.4, but it is important to note that it corresponds with only a 5-fold excess of thiolate being recognized as a solely reactive form. Importantly, as the intracellular concentration of glutathione is at the mM level,^48,49^ the reaction can be considered biologically relevant.

From the data in Figures 6 and S9, equilibrium constants were found to be *K*_eq_^I_1_,AcCys^–^^ = 1.2 × 10^6^ M^–1^, *K*_eq_^I_1_,GS^–^^ = 1.5 × 10^5^ M^–1^, and *K*_eq_^I_1_,Cys^–^^ = 2.6 × 10^4^ M^–1^, which are in close agreement with kinetically determined values (Table 2). The comparison between the *K*_eq_^I_1_,RS^–^^ values revealed that the equilibrium constants are quite sensitive to the structure of the thiolate. The relatively small *K*_eq_^I_1_,Cys^–^^ value compared to the equilibrium constants for the other two thiols indicates that the *k*_off(Cys^–^)_^2^ must be large, which makes *k*_obs(Cys)_^2^ too large to be determined on a stopped-flow instrument. This also explains the low yield of CysNO generation in the applied [Cys]_T_ reported in Figure 1. Based on that, we can postulate that the reversibility of the **I**_**1**_ formation is strongly dependent on the thiolate nature, and the strongest reversibility expressed by *k*_off(RS^–^)_^2^ is expected for NO incorporated in the form of the ferrous-AcMP-11-*S*-nitrosocysteine adduct.

**Table 2:** Equilibrium Constants and Standard Reaction Parameters for the Formation of (AcMP-11)Fe^2+^(N(O)SR) in the Reaction of AcCys, GSH, and Cys with (AcMP-11)Fe^2+^(NO^+^)

	AcCys	GSH	Cys
Δ*H*, kJ/mol	–47 ± 5^a^	nd	nd
Δ*S*, J/mol·K	–53 ± 17^a^	nd	nd
Δ*G* (5 °C), kJ/mol	–31 ± 9^a^ (−32 ± 1^b^)	–29 ± 2^b^	–23 ± 1^b^
*K*_eq_^I_1_^ (5 °C), M^–1^	(1.2 ± 0.7) × 10^6^^c^	(1.5 ± 0.6) × 10^5^^c^	(2.6 ± 0.8) × 10^4^^c^
(kinetic at 5 °C), M^–1^	(1.1 ± 0.7) × 10^6^	(1.5 ± 0.1) × 10^5^	

a Values calculated using activation parameters for the forward and back reactions reported in Table 1.

b Values calculated using ***K***_**eq**_^**I**_**1**_^.

c Values obtained from data reported in Figures 6 and S9.

To compare the generation of **I**_1_ with the three studied thiols, Δ*G*_5°C_ values were calculated from *K*_eq_^I_1_,RS^–^^ or for (AcMP-11)Fe^2+^(N(O)AcCys), Δ*H* and Δ*S* were also estimated from the activation parameters. Reaction parameters estimated for (AcMP-11)Fe^2+^(N(O)AcCys) revealed that its formation is exothermic (Δ*H*_(AcCys^–^)_= −47 kJ/mol) with a favorable free enthalpy (Δ*G*_5°__C(AcCys^–^)_ = −31 kJ/mol). Negative reaction entropy (Δ*S*_(AcCys^–^)_ = −53 J/mol·K) indicates that the reaction will be disfavored with increasing temperatures, thus promoting reversibility. Exothermic reaction character can also be predicted for the other studied thiols (GSH and Cys), but the calculation of the exact values was not possible. The capture of GS^–^ and Cys^–^ by (AcMP-11)Fe^2+^(NO^+^) is also estimated to be quite favorable based on the free enthalpy values of Δ*G*_5°C(GS^–^)_ = −29 kJ/mol and Δ*G*_5°C(Cys^–^)_ = −23 kJ/mol, respectively. These values correlate with the expected impact of the thiol structure on the stability of the **I**_**1**_ intermediate.

### Computational Analysis of *N*- and *S*-Iron-Heme-Coordinated *S*-Nitrosothiols

**Figure 7:**
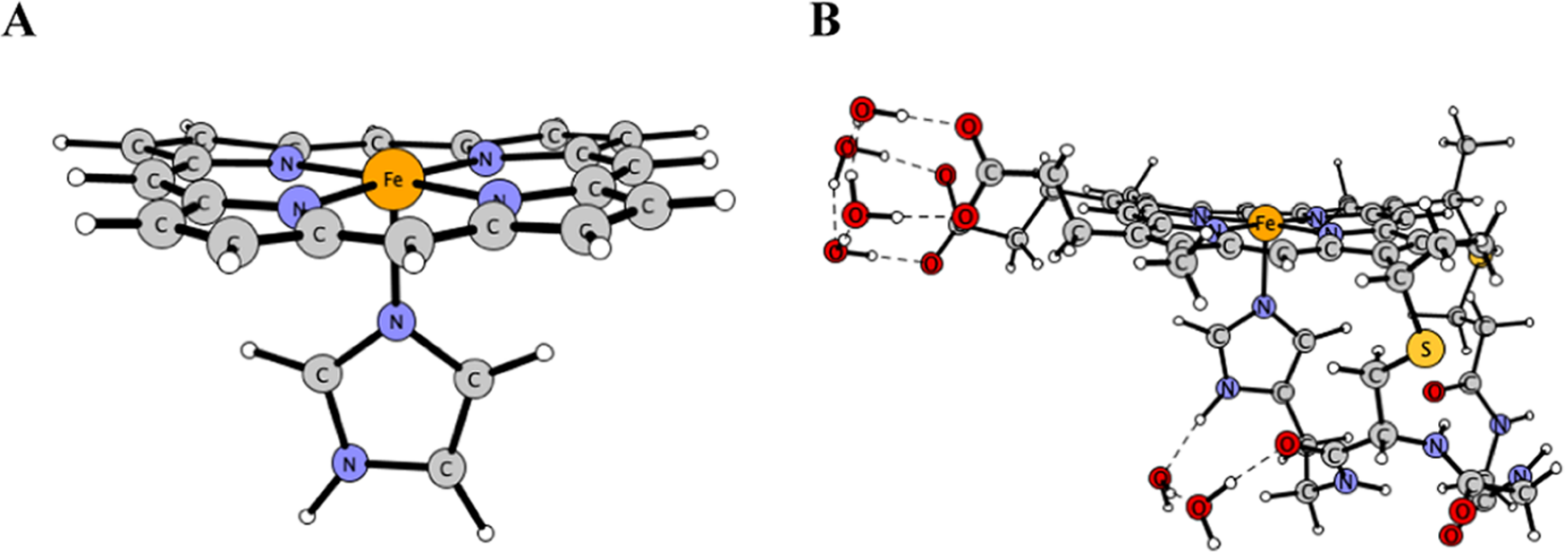
Geometry models of AcMP-11 considered in this study: (a) model 1 and (b) model 2.

The geometry and electronic properties of the complexes, which can be formed in the studied systems were first studied for model 1, followed by the studies of model 2 (Figure 7). The latter considers a larger coordination sphere of the central iron atom but makes slight compromises on the size of the basis set used for computation.

Aqua-, nitroso-, and model-thiol complexes were considered as the starting structures (Figure S10). The Fe–SCH_3_ and Fe–N_im_ bond lengths in Fe(SCH_3_)(Por)(Im) for models 1 and 2 are reported in Table S3. In the next step, the model nitrosothiol complexes (where thiols were represented by the SCH_3_^–^ moiety) were considered (Figure S11). In the case of the system where iron is coordinated by nitrogen, one observes elongation of the Fe–N bond from 1.641 to 1.881 Å (model 1) and from 1.635 to 1.808 Å (model 2), when one goes from Fe(NO)(Por)(Im) to Fe(N(O)SCH_3_)(Por)(Im). This is accompanied by only minor changes in the length of the axial Fe–imidazole bond (Table S3). The sulfur–nitrogen bond length is equal to 1.771 Å in model 1 and 1.894 Å in model 2, which is slightly more than in the isolated SCH_3_NO species (1.768 Å). In the alternative complex in which the nitrosothiol is bound via the sulfur atom, the Fe–S bond is also longer (2.424 Å—model 1, 2.385 Å—model 2) than in Fe(SCH_3_)(Por)(Im) (2.243 Å—model 1, 2.248 Å—model 2). By contrast, the axial Fe–N_im_ bond is shortened from 2.078 to 1.990 Å (model 1) and from 2.015 to 1.971 Å (model 2) in Fe(SCH_3_NO)(Por)(Im). In the case of this nitrosothiol system, the N–S bond length is longer (1.834 Å in model 1 and 2.048 Å in model 2) than in the previously discussed case.

The comparison of the formation energy of the structures coordinated by nitrogen and sulfur atoms reveals that the more stable structure is formed when the Fe^3+^ ion is bound directly via the nitrogen (the difference in stability between the two structures amounts to −10.4 kcal/mol, model 1 and −14.2 kcal/mol, model 2) (Table S4).

**Figure 8:**
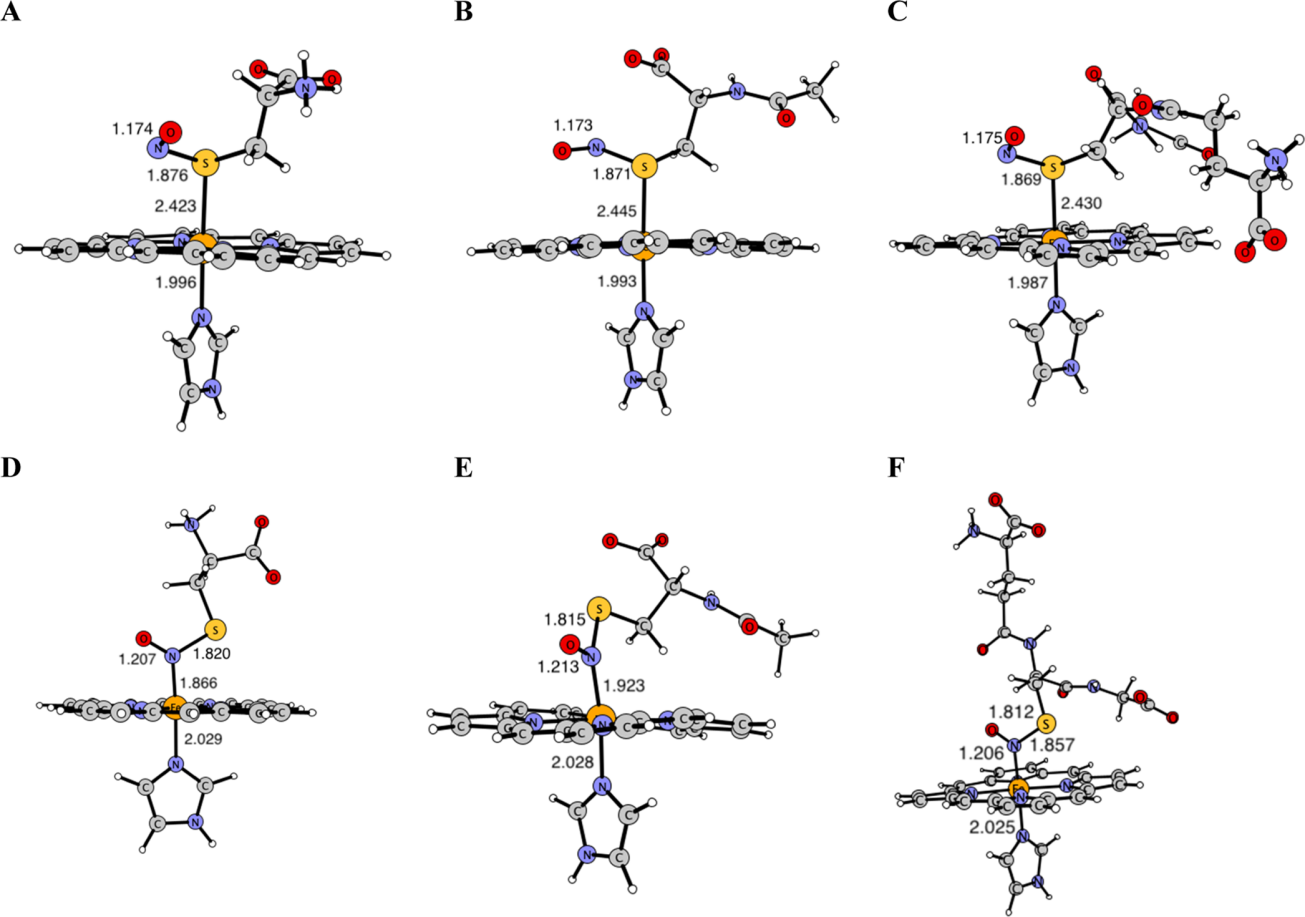
Geometry structures of model 1 with entire thiol moiety of Cys, AcCys, and GSH with selected bond lengths (in Å); *S*-bound nitrosothiol complexes of Cys (A), AcCys (B), and GSH (C) and *N*-bound nitrosothiol complexes of Cys (D), AcCys (E), and GSH (F).

In the next step, the nitrosothiol moiety was further enlarged to represent entire molecules of Cys, AcCys, and GSH to study the differences between the examined nitrosothiols—see Figure S12 for the resulting structures. The type of the thiol influences both bonds formed by the iron ion. The Fe–S bond length changes in the following order: Fe–AcCys < Fe–Cys < Fe–GSH, while the Fe–N_im_ bond length follows the reversed order: Fe–N_im_ in the AcCys complex > Fe–N_im_ in the Cys complex > Fe–N_im_ in the GSH complex (Figure 8, Table S3). In the case of the nitrosothiol systems in which iron is coordinated by the sulfur atoms (Figure 8, Table S3), the Fe–S bond length is the shortest in the CysNO complex, followed by the GSNO one, and the longest in the AcCysNO complex (Table S3). The Fe–N_im_ bond lengths change in a different order; it is the longest in the CysNO complex and the shortest in the GSNO one (Table S3). The S–N bond length in the iron-coordinated structures is the longest in the CysNO complex (1.876 Å). It is slightly shorter (1.871 and 1.869 Å) in AcCysNO and GSHNO, respectively, but the differences in bond lengths of these nitrosothiols are almost negligible. In each structure, the S–N bond is elongated in comparison with the isolated nitrosothiol molecules (Figure 8, Table S3). The N–O bond lengths are comparable in all three complexes.

When the nitrosothiol moiety is bound to the iron atom by the nitrogen atom, the Fe–N bond length changes in the following order: Fe–NOGS < Fe–NOCys < Fe–NOAcCys. The axial Fe–N_im_ bond is almost invariant with respect to the type of thiol (Table S3). The N–S bond length is the shortest in the GSNO system (1.812 Å), intermediate in the AcCysNO complex (1.815 Å), and the longest in the CysNO one (1.820 Å). The N–S bond elongation (as compared to the N–S bond length in the isolated CysNO and AcCysNO nitrosothiols) is smaller (ca. 0.01 Å) than in the complexes where the nitrosothiols are bound to the iron atom via the sulfur atom (ca. 0.07 Å) (Figure 8). This may indicate that the latter structures are more eager to decompose to NO and thiol, serving as NO-delivering species. In the case of the GSNO structure, the N–S bond is elongated in the *S*-bound structure by 0.04 Å, while shortened by 0.02 Å in the N-bound one. Finally, examining the N–O bond length shows that these are comparable in all studied *S*-nitrosothiols (1.206–1.213 Å) (Figure 8, Table S3).

As in the model complexes, the structures in which the nitrosothiols are bound via the N-atom are more stable than the isomers bound via the S-atom (Table S4). The energy difference is the largest in the case of CysNO (9.2 kcal/mol) and the smallest in the case of AcCysNO (0.5 kcal/mol). In the case of the GSNO, it amounts to 4.1 kcal/mol.

The coordination of nitrosothiols via the nitrogen atom is energetically privileged over the coordination via the sulfur atom, which is found irrespective of the model used for the calculations; however, both isomeric forms can exist. The observed differences in the N–S bonds between the complexes where the nitrosothiols are bound to the iron atom via sulfur atoms or nitrogen atoms suggest that the S-bonded structures are more eager to decompose to NO and thiol. Stronger S–N bonds in complexes coordinating *S*-nitrosothiols via N-atoms are in line with kinetic results and the proposed *N*-coordinated (AcMP-11)Fe^2+^(N(O)SR) complex as an intermediate releasing free RSNO species.

## Summary and Conclusions

Two reaction pathways potentially leading to the generation of low-molecular-weight RSNO species from NO and thiols with the assistance of *N*-acetylmicroperoxidase-11 have been tested. One of the pathways assumes an NO attack on the Fe^3+^(RS) moiety, whereas the second one assumes RS^–^ binding to the Fe^2+^–NO^+^. Efficient AcMP-11-mediated transfer of NO to biologically important thiols, glutathione, cysteine, and *N*-acetylcysteine, leading to the generation of corresponding *S*-nitrosothiols (GSNO, CysNO, and AcCysNO) has been confirmed in both experimental approaches. The yield of the *S*-nitrosothiol formation depends on the concentration of both NO and thiol, requiring an excess of both over AcMP-11 to achieve maximum efficiency, limited by the concentration of (AcMP-11)Fe^3+^(H_2_O). Time-resolved spectroscopic studies on the reaction of (AcMP-11)Fe^2+^(NO^+^) with RS^–^ and the reaction of (AcMP-11)Fe^3+^(RS) with NO under inert conditions revealed that, in both cases, a double-step transformation process leading to the accumulation of (AcMP-11)Fe^2+^(NO) occurs. The intermediate with the same UV–vis spectral features was registered in both reactivity scenarios and was assigned to the *N*-coordinated *S*-nitrosothiol, (AcMP-11)Fe^2+^(N(O)SR). Transformation of (AcMP-11)Fe^2+^(N(O)SR) to inert (AcMP-11)Fe^2+^(NO) seems to be crucial for the generation of free *S*-nitrosothiols, preventing their decomposition via shutting down their coordination to the Fe^2+^ center. The activity of AcMP-11 toward oxidative coupling of RS and NO can be restored by simple reoxidation of (AcMP-11)Fe^2+^(NO) to the ferric-AcMP-11, making RSNO generation catalytic.

Kinetic studies on the formation of iron-coordinated *S*-nitrosothiol supported by theoretical analysis unraveled the molecular mechanism of (AcMP-11)Fe^2+^(N(O)SR) formation. Among two possible reactivity pathways, nucleophilic attack of RS^–^ on (AcMP-11)Fe^2+^(NO^+^) is kinetically and energetically much more favorable. Operation of the second potentially feasible mechanism, NO attack on (AcMP-11)Fe^3+^(RS) was excluded. Theoretical calculations revealed that the *N*-coordination of the *S*-nitrosothiol to the heme-iron strengthens the S–N bond compared to *S*-coordination, providing support for the kinetically determined mechanism. This indicates that the *N*-coordination mode should operate in a pathway of the *S*-nitrosothiol formation mechanism on iron-heme sites, whereas *S*-coordination one promotes their decomposition. Kinetic studies on the RS^–^ capture by (AcMP-11)Fe^2+^(NO^+^) revealed a reversible formation of the (AcMP-11)Fe^2+^(N(O)RS) intermediate occurring solely in the reaction with thiolate. The reversibility of (AcMP-11)Fe^2+^(N(O)SR) formation assessed by “off” reaction depends on the thiol structure and is assumed to be the most effective for (AcMP-11)Fe^2+^(N(O)Cys).

Our study suggests that heme proteins may be a part of a reversible interconversion of NO in a form of an iron-coordinated RSNO motif. This reversibility may be critically important for the regulation of the NO bioavailability with the iron-heme centers in nature. The reported study deepens the understanding of the ferric-heme-assisted storage of NO in the form of *S*-nitrosothiols, as well as provides insight into the molecular mechanism and factors facilitating RSNO generation. Our research also contributes to the comprehension of the double role of the heme centers in NO signaling through the modulation of biological NO concentration (RSNO generation and decomposition) providing additional support to the crucial role of the RSNO coordination mode in determining the S–N bond strength.

## Supporting Information Available

Additional figures and tables describing experimental and computational details (PDF)
