# Supplementary material for: Unraveling the Molecular Mechanism of S-Nitrosation Mediated by N-Acetylmicroperoxidase-11
Source: Inorg Chem. 2023 Mar 30;62(14):5630–43. doi: 10.1021/acs.inorgchem.3c00180 (PMC10091411; doi:10.1021/acs.inorgchem.3c00180)
Supplement: Supplementary file 1 — ic3c00180_si_001.pdf [file ic3c00180_si_001.pdf]

Supporting Information

Unraveling the molecular mechanism of S-nitrosation mediated by N-acetylmicroperoxidase-11

Maria Oszajca[a*], Angelika Jodłowska[a], Dorota Rutkowska-Zbik[b], Konrad Kieca[a,c], Grażyna Stochel[a]

maria.oszajca@uj.edu.pl

[a]*Jagiellonian University, Faculty of Chemistry, 30-387 Krakow, Poland*

[b]*Polish Academy of Sciences, Jerzy Haber Institute of Catalysis and Surface Chemistry, 30-239 Krakow, Poland*

[c]*Jagiellonian University, Doctoral School of Exact and Natural Sciences, 30-348 Krakow, Poland*

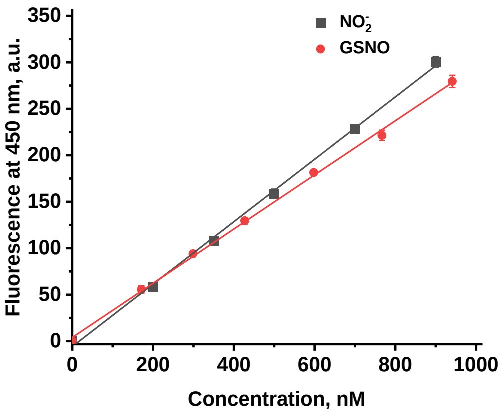

Figure S1. GSNO standard calibration curve for the fluorescence-based determination of S-nitrosothiol concentration (red). Each calibration point represents the fluorescence difference between the GSNO sample treated with $HgCl_2$ and untreated. For comparison, an analogous standard calibration curve obtained for NO_2^- has been presented (black). Good agreement of these two calibration curves confirms the lack of interferences of $HgCl_2$ in the recorded fluorescence values of 2,3-naphthotriazole. Experimental conditions: [HCl] = 0.25 mM, [2,3-diaminonaphthalene] = 54 mM, [$HgCl_2$] = 0.17 mM, [NaOH] = 0.12M.

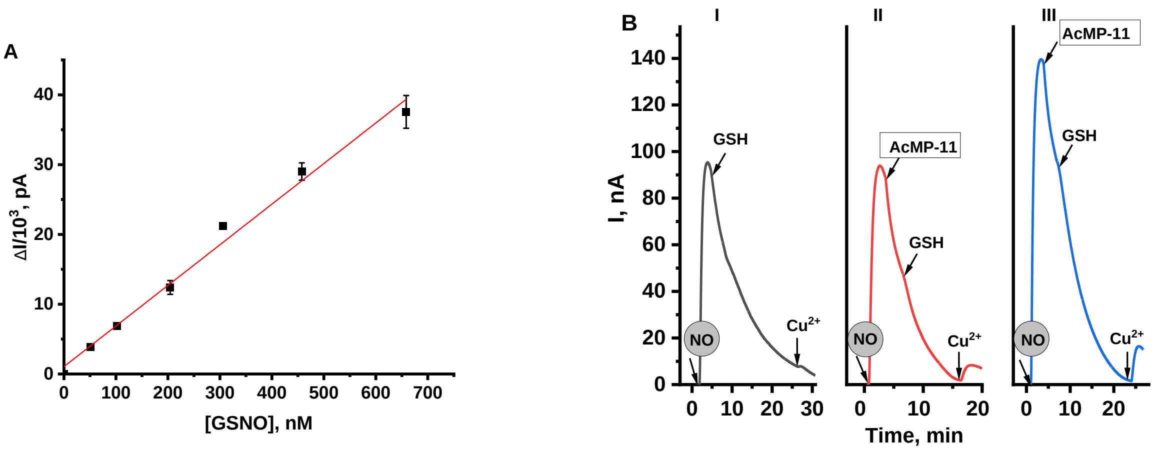

Figure S2. (A) Calibration curve for electrochemical analysis of the amount of generated S-nitrosoglutathione (GSNO) obtained with the application of selective NO sensor. Experimental conditions: [Tris buffer] = 0.05 M, pH = 7.4; $[Cu^{2+}]$ = 1 mM; [ascorbic acid] = 5 μM. (B) Amperometric detection of GSNO generation. Representative recordings by NO electrode illustrating GSNO generation from GSH and NO in the presence of AcMP-11. When the electrode response reached the maximum after NO introduction, (I) GSH only or (II) and (III) AcMP-11 and GSH were added, followed by Cu^{2+} addition after the current drop. Applied excess of $[GSH]_T$ in the reaction mixture provided sufficient concentration for the reduction of Cu^{2+} to Cu^{+} which is recognized as a GSNO decomposing species. Experimental conditions: [AcMP-11] = 1 μM, $[GSH]_T$ = 50 μM, $[Cu^{2+}]$ = 500 μM, [NO] = 400 nM (I-II); 550 nM (III), [phosphate buffer] = 0.025 M, pH = 7.4, T = 25 °C. $[GSH]_T$ is the total concentration of glutathione.

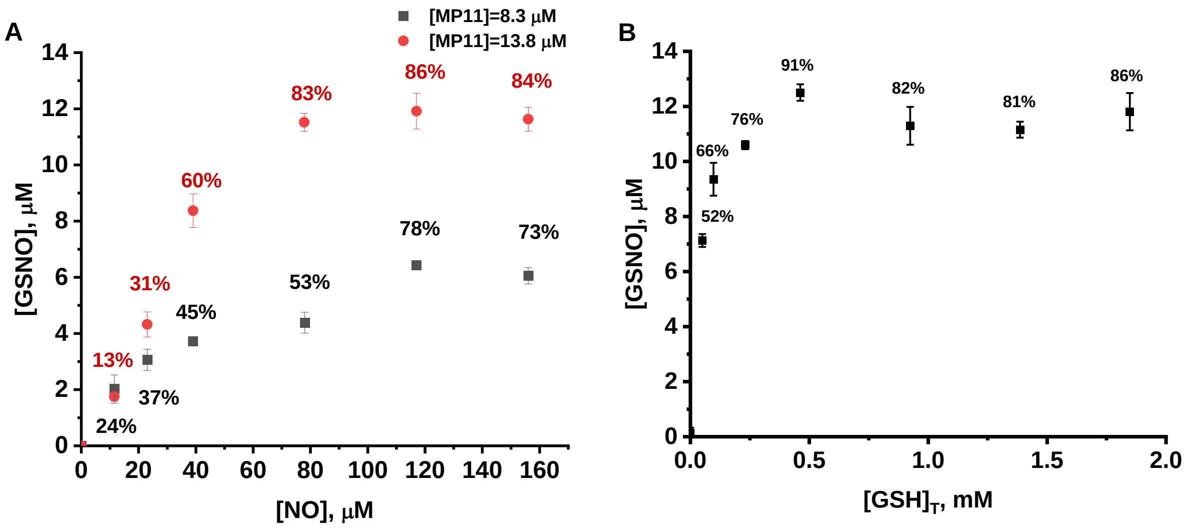

Figure S3. S-nitrosoglutathione (GSNO) formation in the reaction of (AcMP-11)Fe^{3+}(GS) with NO. (A) NO-dependent formation of GSNO in the presence of 1.38 mM GSH and either 13.8 μM AcMP-11 (red circles) or 8.3 μM AcMP-11 (black squares). (B) GSH concentration-dependent formation of GSNO in the presence of 78 μM [NO] and 13.8 μM AcMP-11. Experimental conditions: [phosphate buffer] = 0.025 M pH = 7.4, [DTPA] = 0.25 mM.

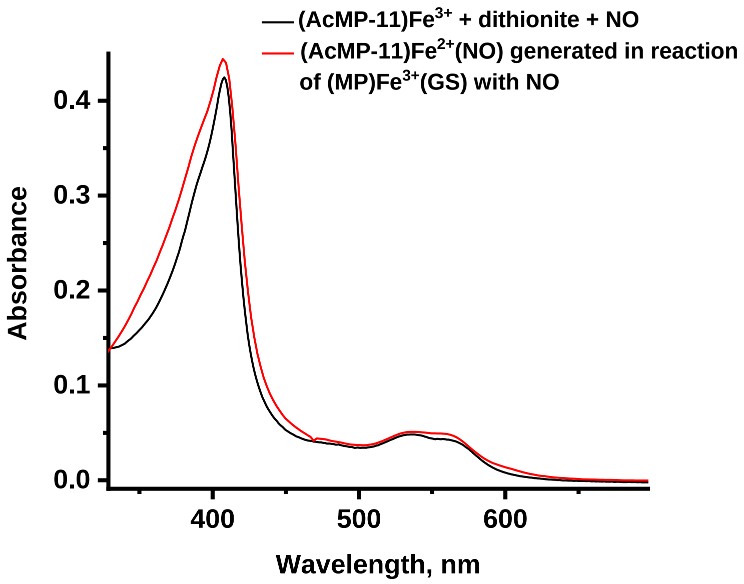

Figure S4. UV-vis spectra of (AcMP-11Fe^{2+}(NO) generated in the reaction of (AcMP-11Fe^{3+}(GS) with NO (red) and the spectrum of the same product obtained in the reaction of (AcMP-11)Fe^{2+} with NO (black) for comparison. Experimental conditions: [AcMP-11] = 5 μM, [GSH] = 0.5 mM, [NO] = 0.25 mM [phosphate buffer] = 0.025 M, pH =7.4, [DTPA] = 0.25 mM, [sodium dithionite] = 75 μM (for (AcMP-11)Fe^{3+} reduction).

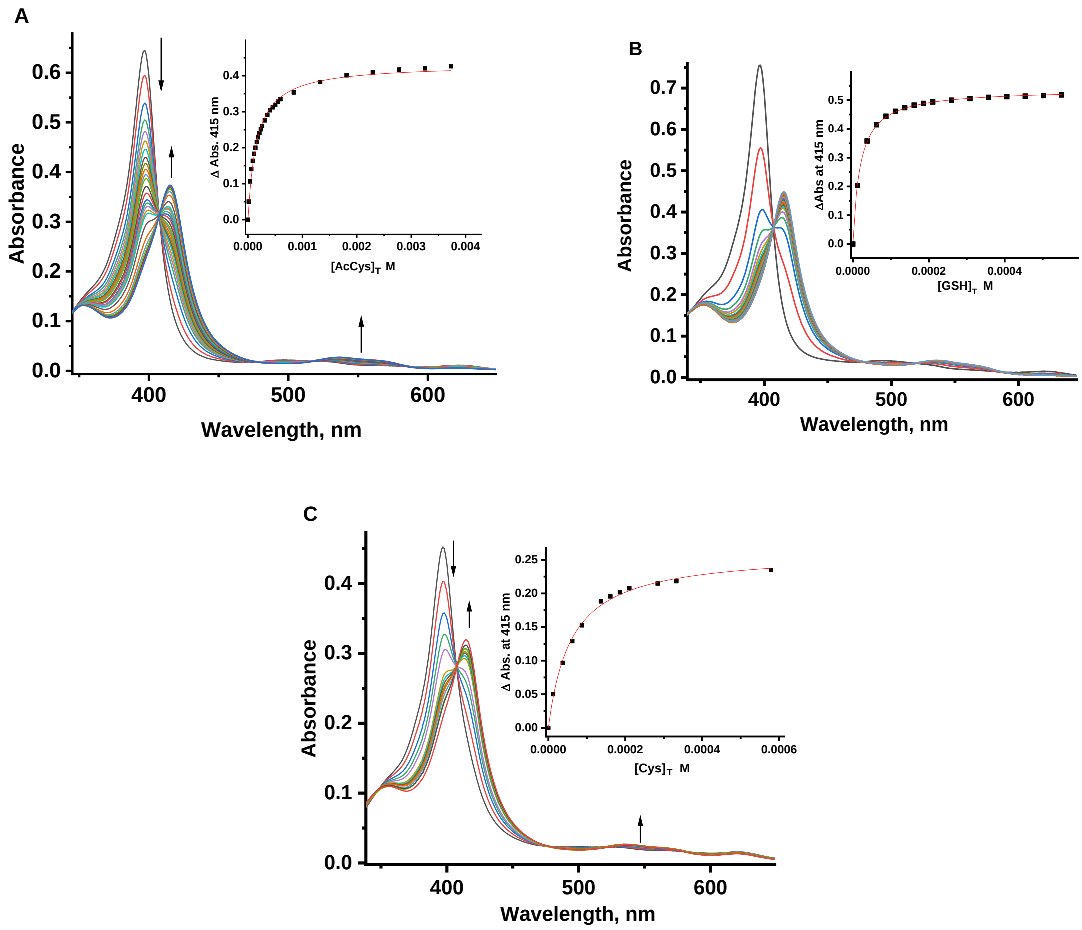

Figure S5. UV-Vis spectra of AcMP-11 registered in the presence of various concentrations of $[RSH]_T$ (A) -AcCys, (B) GSH, (C) Cys. Insets illustrate absorbance change at 415 nm vs $[RS]_T$. Experimental conditions: [phosphate buffer] = 0.1 M, pH = 7.4, [DTPA] = 0.25 mM, T = 20 °C, anaerobic conditions were applied to avoid (AcMP-11)Fe^{3+}(RS) decomposition occurring in aerated solution.

To complete the kinetic picture of the studied system, which was necessary for further data analysis, we have performed kinetic studies on the formation of (AcMP-11)Fe^{3+}(RS) (**Figure S6)**. The determined rate constants and related kinetically determined apparent equilibrium constants are reported in **Table S1**.

Table S1. Rate and apparent equilibrium constants for the reversible binding of RS to AcMP-11 at pH = 7.4 and T = 20 °C.

	GSH	Cys	AcCys
$k_{\mathrm{on}}^{\mathrm{RS}}$, $M^{-1}s^{-1}$	$(1.3 \pm 0.1) \times 10^6$	$(5.5 \pm 0.1) \times 10^5$	$(1.1 \pm 0.1) \times 10^6$
$k_{\mathrm{off}}^{\mathrm{RS}}$, s^{-1}	27 ± 4	26 ± 1	182 ± 6
$K_{\mathrm{app}}^{\mathrm{RS}}$, M^{-1}	$(4.6 \pm 0.8) \times 10^4$	$(2.1 \pm 0.1) \times 10^4$	$(6.0 \pm 0.6) \times 10^3$
$K_{\mathrm{app}}^{\mathrm{RS,th}}$, M^{-1}	$(5.2 \pm 0.1) \times 10^4$	$(1.7 \pm 0.1) \times 10^4$	$(6.2 \pm 0.2) \times 10^3$

$K_{\mathrm{app}}^{\mathrm{RS}}$– kinetically determined values: k_1/k_{-1}

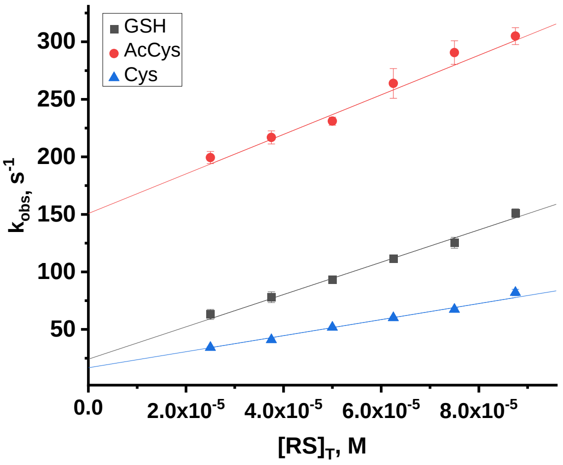

Figure S6. Linear dependence of k_{obs} on $[RS]_T$ for the reaction of thiolates with (AcMP-11)Fe^{3+}(H_2O) at pH 7.4. Experimental conditions: [AcMP-11] = 2.5 × 10^{-5} M, $[RS]_T$ = GSH, AcCys, Cys, [phosphate buffer] = 0.1 M, pH = 7.4, [DTPA] = 0.25 mM, T = 20 °C, anaerobic conditions were applied to avoid (AcMP-11)Fe^{3+}(RS) decomposition occurring in aerated solution.

Determination of AcCys pK_a at various temperatures

pK_a of the –SH group in AcCys was determined at 5, 10, 15, 20, and 25 °C spectrophotometrically (Lambda 950 PerkinElmer) by measuring AcCys spectra in the set of buffer solutions at pH range 7-11.5. The spectra were measured in a wavelength range between 200 and 350 nm. Due to the instability of AcCys in solution, classical pH potentiometric titration was abandoned. To cover the studied pH range 0.01 M Tris, CHES, and CAPS buffers were prepared. The ionic strength of all buffer solutions was kept constant (I = 0.26 M NaCl - the ionic strength was adjusted to the ionic strength of the buffer solutions used in the kinetic measurements). A freshly prepared stock solution of AcCys was added to 2ml of buffer solution set to a selected pH value. Each tested buffer solution was left to equilibrate at an appropriate temperature for 15 min before AcCys addition and the spectra measurement. For each buffer solution, the corrections for the temperature at which the buffer was used have been introduced. **Figure S7A** shows representative spectroscopic data of AcCys at different pH values at 20 °C, while **Figure S7B** shows the usual pH dependence of the absorption at 240 nm. Experimental data were fitted to the sigmoidal equation (OriginPro 2019: DoseResp function) to evaluate the pK_a according to the equation:

$y = A_{min} + \frac{A_{max}-A_{min}}{1+10^{(\log x0 - x)*p}}$, where y is the absorbance at the selected wavelength registered at a particular pH, A_{min} and A_{max} represent the corresponding minimum and maximum absorbance, $\log x_0$ = pK_a represents the center of the Hill slope, and *p* is the Hill coefficient of the slope obtained from the plot measured at the inflection point. p*K*a values reported in **Table S2** are mean values determined in two independent experiments.

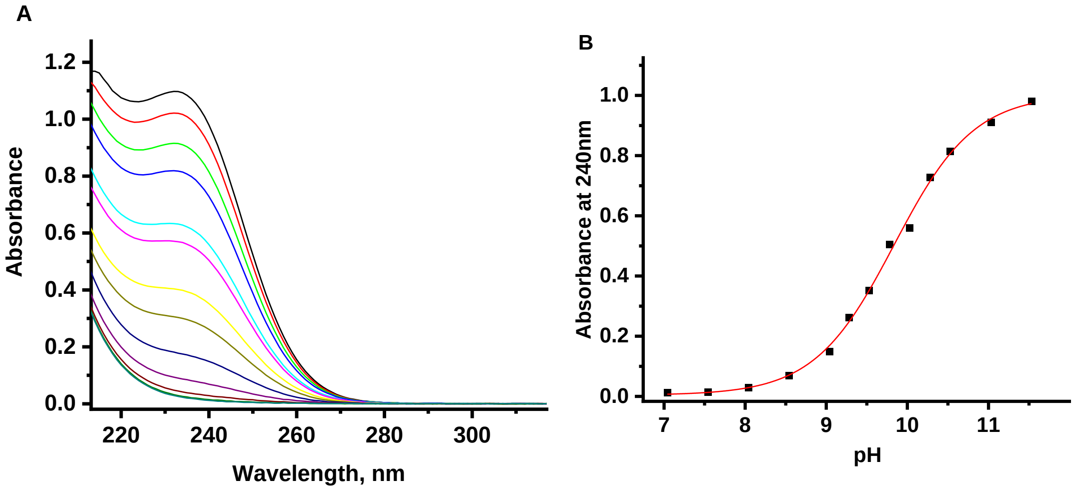

Figure S7. (A) Representative spectra of AcCys registered at various pH values at 20.0 °C. (B) Absorbance change at 240 nm at the selected pH range. Experimental conditions: [AcCys] = 0.1 mM, [buffer] = 0.01M, pH 7.0-9.0 Tris buffer, 9.2-9.7 CHES buffer, 10.0-11.5 CAPS buffer, I = 0.26 M NaCl, T = 20.0 °C.

Table S2. pK_a values of –SH group of AcCys determined at selected temperatures and I = 0.26 M (NaCl).

***T*, °C**	**p*K*a**	
	**AcCys**	**GSH**
5.0	9.95 ± 0.02	9.32 ± 0.02
10.0	9.88± 0.04	
15.0	9.80 ± 0.04	
20.0	9.75 ± 0.02	
25.0	9.70 ± 0.03	

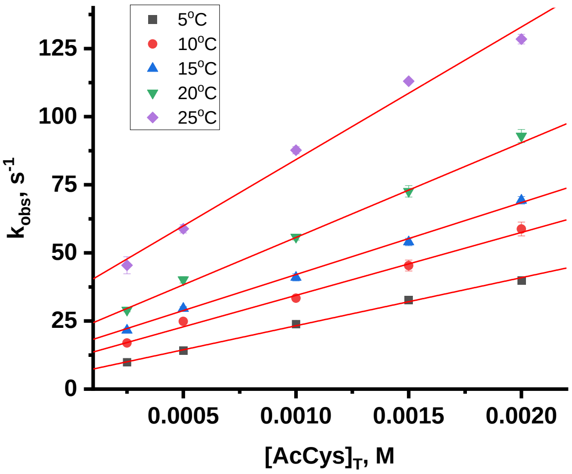

Figure S8. Dependences of $k^2_{\mathrm{obs(AcCys)}}$ in a function of $[\mathrm{AcCys}]_T$ for the reaction of (AcMP-11)Fe^{2+}(NO^+) with AcCys measured at various temperatures: 5°C, 10°C, 15°C, 20°C, 25°C. Experimental conditions: [AcMP-11] = 5 × 10^{-6} M, [phosphate buffer] = 0.1 M, [NO] = 1.8 × 10^{-4} M, [DTPA] = 0.25 mM.

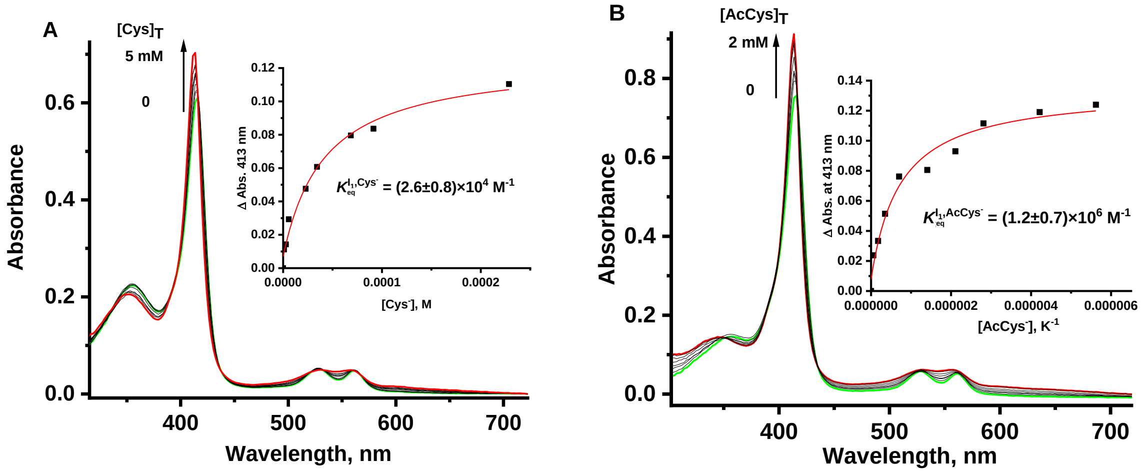

Figure S9. UV-Vis spectra of (AcMP-11)Fe^{2+}(RSNO) registered in the presence of various RS concentrations and constant [NO]. (A) (AcMP-11)Fe^{2+}(CysNO), (B) (AcMP-11)Fe^{2+}(AcCysNO). Experimental conditions: [MP-11] = 5 × 10^{-6} M, [NO] = 1.8 × 10^{-4} M, [phosphate buffer] = 0.1 M, [DTPA] = 0.25 mM, pH = 7.4, T = 5 °C.

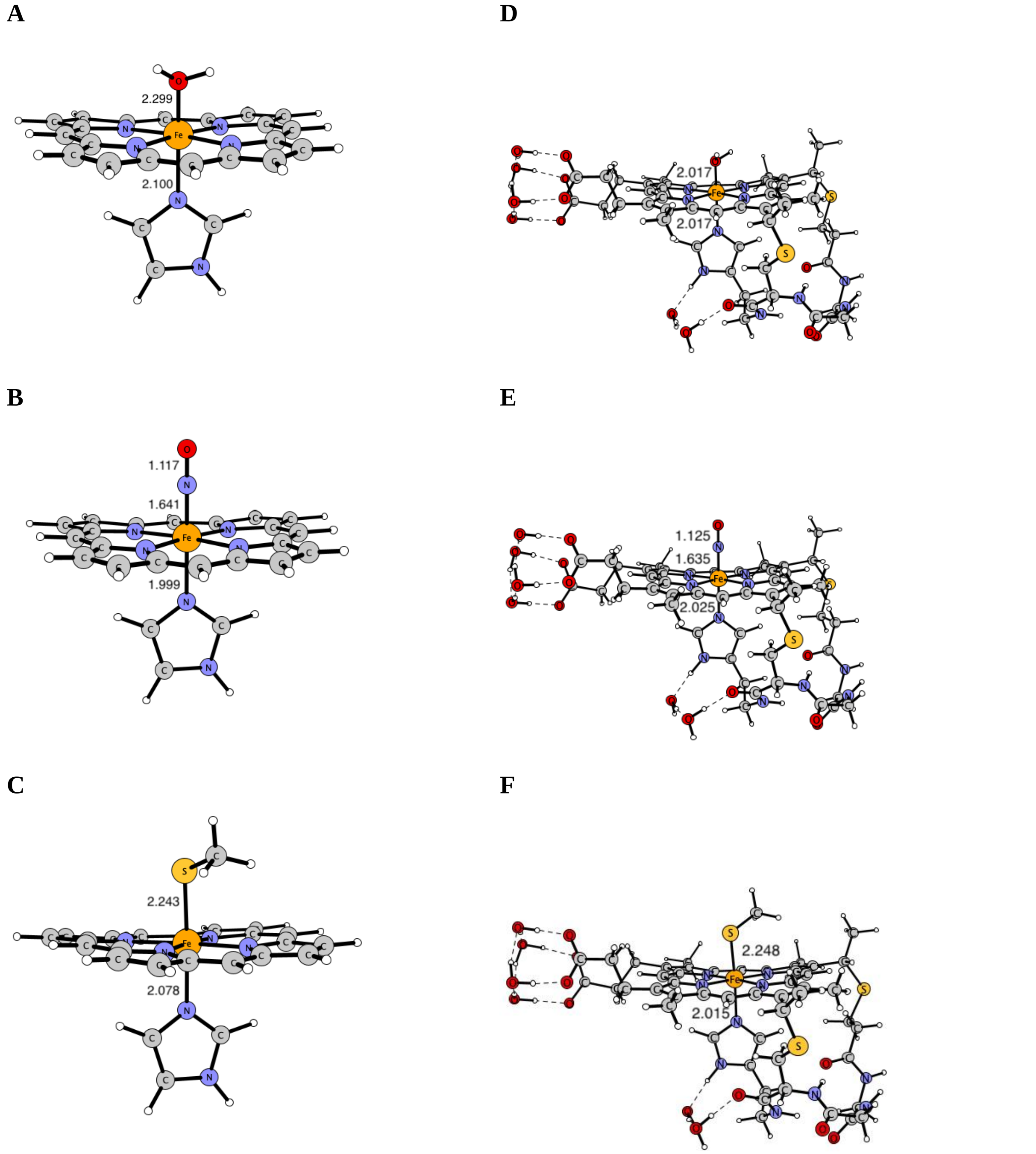

Figure S10. Geometry structures considered in this study: with selected bond lengths (in Å): aqua complexes (A and D), nitrosyl complexes (B and E), thiol complexes (C and F); model 1- (A, B and C) model 2 (D, E, and F).

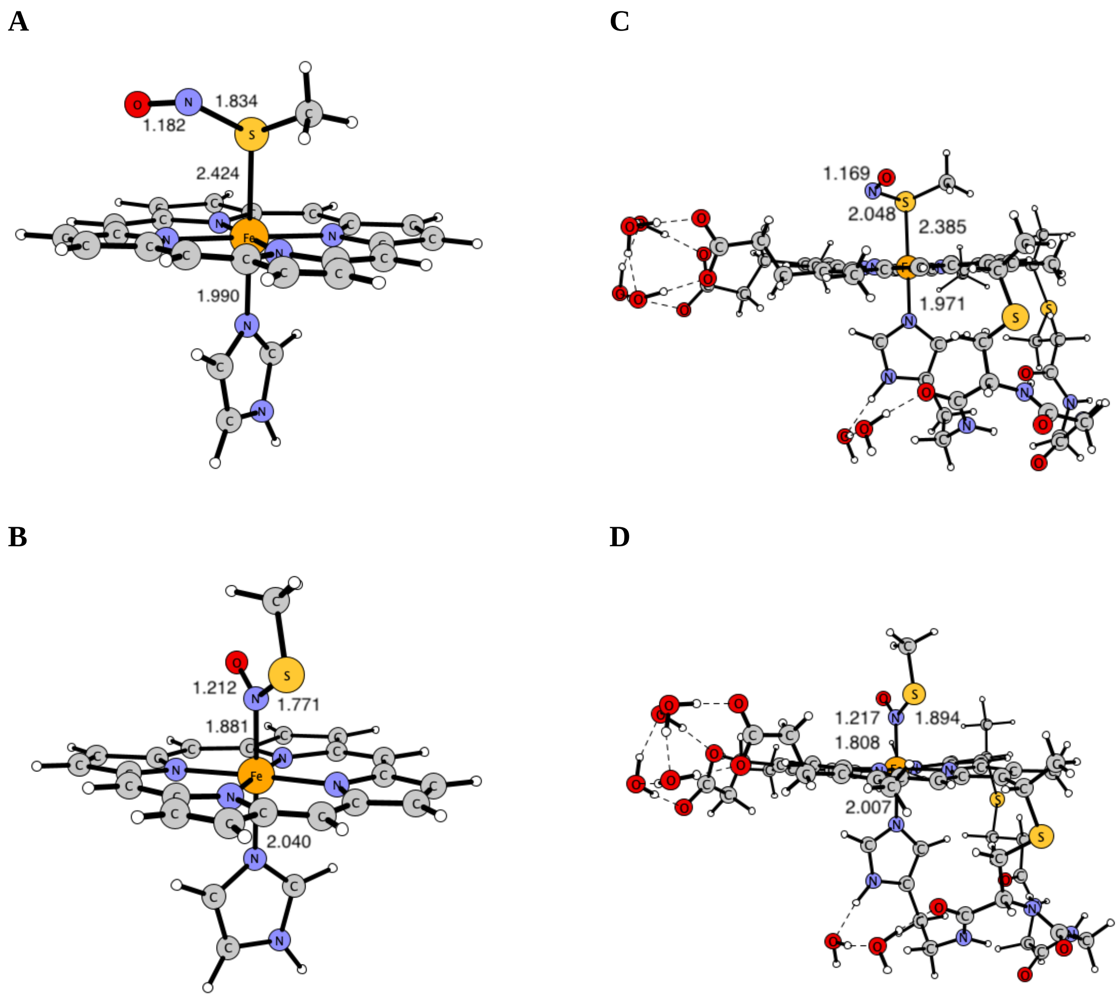

Figure S11. Geometry structures considered in this study: with selected bond lengths (in Å): S-bound nitrosothiol complexes (A and C), N-bound nitrosothiol complexes (B and D), model 1- (A, B) model 2 (C, D).

A **B**

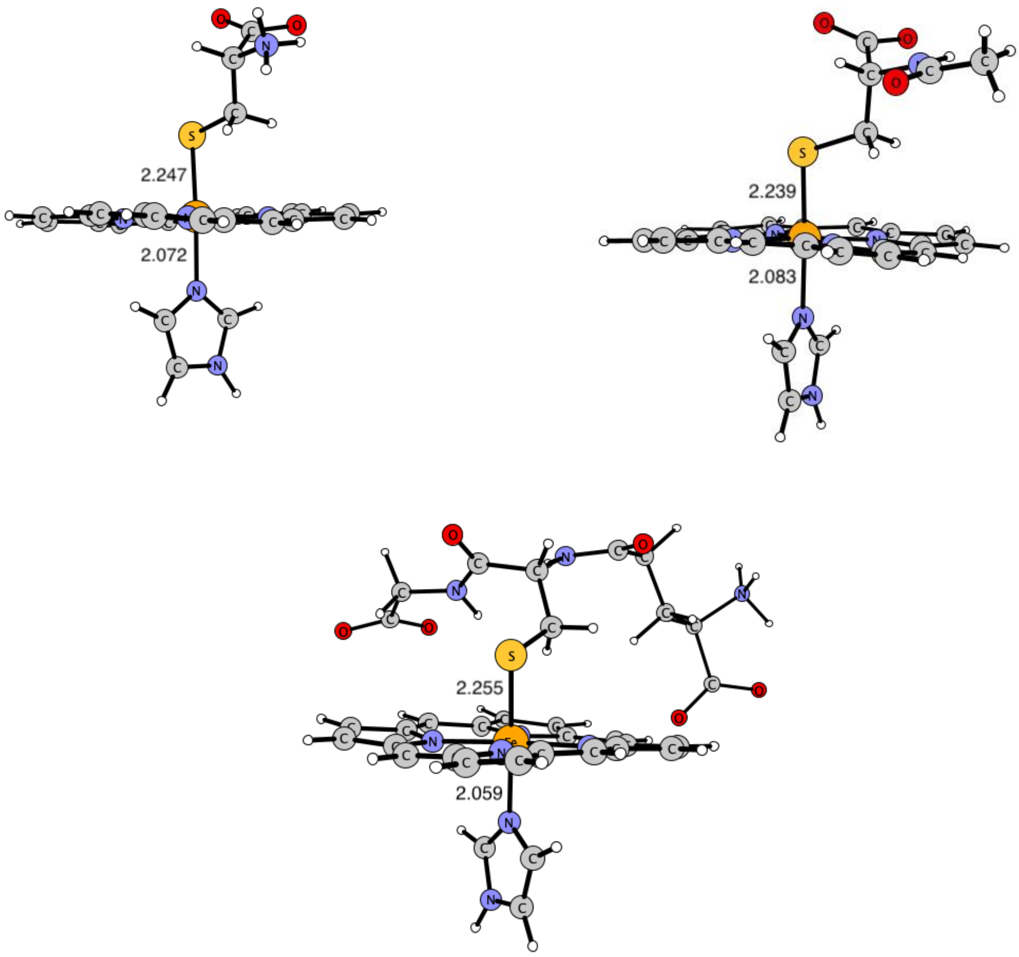

Figure S12. Geometry structures of model 1 with entire thiol moiety: Cys (A), AcCys (B), GSH (C) with selected bond lengths (in Å).

Table S3. Selected bond lengths in [Å].

System	model	Fe-N	Fe-S	N-O	S-N	Fe-N$_{im}$
**(Por)Fe(NO)(Im)**	1	1.641, (1.635)	-	1.117	-	1.999
	(2)			(1.125)		(2.025)
**(Por)Fe(SCH$_3$)(Im)**	1	-	2.242	-	-	2.078
	(2)		(2.248)			(2.015)
**(Por)Fe(Cys)(Im)**	1	-	2.247	-	-	2.072
**(Por)Fe(AcCys)(Im)**	1	-	2.239	-	-	2.083
**(Por)Fe(GS)(Im)**	1	-	2.255	-	-	2.059
**SCH$_3$NO**	-	-	-	1.195	1.768	-
**CysNO**	-	-	-	1.190	1.810	-
**AcCysNO**	-	-	-	1.194	1.802	-
**GSNO**	-	-	-	1.184	1.831	-
**(Por)Fe(S(NO)CH$_3$)(Im)**	1	-	2.424	1.182	1.834	1.990
	(2)		(2.385)	(1.169)	(2.048)	(1.971)
**(Por)Fe(S(NO)Cys)(Im)**	1	-	2.423	1.174	1.876	1.996
**(Por)Fe(S(NO)AcCys)(Im)**	1	-	2.445	1.173	1.871	1.993
**(Por)Fe(S(NO)GS)(Im)**	1	-	2.430	1.175	1.869	1.987
**(Por)Fe(N(O)SCH$_3$)(Im)**	1	1.881	-	1.212	1.771	2.040
	(2)	(1.808)		(1.217)	(1.894)	(2.007)
**(Por)Fe(N(O)Cys)(Im)**	1	1.866	-	1.207	1.820	2.029
**(Por)Fe(N(O)AcCys)(Im)**	1	1.923	-	1.213	1.815	2.028
**(Por)Fe(N(O)GS)(Im)**	1	1.857	-	1.206	1.812	2.025

Table S4. DFT:B3LYP energy differences (ΔE) between the nitrosothiols coordinated by nitrogen (E_N) and sulphur (E_S) atoms to theoretical models of the AcMP-11.

**theoretical model**	**nitrosothiol**	**Δ E = E_N -E_S [kcal/mol]**
model 1	SCH$_3$NO	-10.4
	CysNO	-9.2
	AcCysNO	-0.5
	GSNO	-4.1
model 2	SCH$_3$NO	-14.2
